# Evolution and Functional Divergence of *SUN* Genes in Plants

**DOI:** 10.3389/fpls.2021.646622

**Published:** 2021-03-08

**Authors:** Li Yuan, Jingwen Pan, Shouhong Zhu, Yan Li, Jinbo Yao, Qiulin Li, Shengtao Fang, Chunyan Liu, Xinyu Wang, Bei Li, Wei Chen, Yongshan Zhang

**Affiliations:** ^1^State Key Laboratory of Cotton Biology, Institute of Cotton Research, Chinese Academy of Agricultural Sciences, Anyang, China; ^2^College of Plant Science, Tarim University, Xinjiang, China; ^3^Zhengzhou Research Base, State Key Laboratory of Cotton Biology, Zhengzhou University, Zhengzhou, China

**Keywords:** SUN proteins, evolution, divergence, cotton, reproductive development

## Abstract

SUN-domain containing proteins are crucial nuclear membrane proteins involved in a plethora of biological functions, including meiosis, nuclear morphology, and embryonic development, but their evolutionary history and functional divergence are obscure. In all, 216 SUN proteins from protists, fungi, and plants were divided into two monophyletic clades (Cter-SUN and Mid-SUN). We performed comprehensive evolutionary analyses, investigating the characteristics of different subfamilies in plants. Mid-SUNs further evolved into two subgroups, SUN3 and SUN5, before the emergence of the ancestor of angiosperms, while Cter-SUNs retained one subfamily of SUN1. The two clades were distinct from each other in the conserved residues of the SUN domain, the TM motif, and exon/intron structures. The gene losses occurred with equal frequency between these two clades, but duplication events of Mid-SUNs were more frequent. In cotton, SUN3 proteins are primarily expressed in petals and stamens and are moderately expressed in other tissues, whereas SUN5 proteins are specifically expressed in mature pollen. Virus-induced knock-down and the CRISPR/Cas9-mediated knockout of *GbSUN5* both showed higher ratios of aborted seeds, although pollen viability remained normal. Our results indicated divergence of biological function between SUN3 and SUN5, and that SUN5 plays an important role in reproductive development.

## Introduction

The nuclear envelope (NE) provides physical rigidity to the nucleus, protects the genome, organizes chromatin, functions in meiotic chromosome pairing, and positions the nucleus within the cell (Starr and Fridolfsson, 2010). The NE, consisting of an outer nuclear membrane (ONM) that is closely associated with perinuclear endoplasmic reticulum (ER) and an inner nuclear membrane (INM) that is connected to the ONM via the nuclear pores, plays a vital role in regulating transport into and out of the nucleus. The NE is also involved in the physical positioning of the nucleus and in the processes of cell division and nucleo-cytoplasmic signaling (Graumann et al., 2010). Significant progress has now been made in the study of novel plant NE proteins. These proteins include a Linker of Nucleoskeleton and Cytoskeleton (LINC) complex based on INM Sad1/Unc-84 (SUN)-domain proteins, ONM Klarsicht/ANC-1/Syne-1 Homology (KASH) proteins, and nuclear lamina associated proteins (CRWNs, KAKU4, and NEAPs) (Poulet et al., 2017). Evolution of KASH domain proteins has resulted in increasing complexity; some are highly conserved and appear in all species, but others are restricted in distribution (Zhou and Meier, 2014; Poulet et al., 2017). Nuclear lamina associated proteins present in plants but is absent in unicellular species, which may attribute to plants evolved a lamina-like structure (Poulet et al., 2017). However, SUN proteins appear throughout and may be one of the earliest evolving components of the plant NE (Murphy et al., 2010; Graumann et al., 2014; Poulet et al., 2017). This suggesting that SUN domain proteins are essential for most organisms. SUN-domain proteins are INM proteins that are part of the linker cytoskeletal elements with the nucleoskeleton (LINC) complexes (Crisp et al., 2006; Starr and Fridolfsson, 2010). SUN proteins are conserved in non-plant and plant systems and have evolved into Cter-SUN and Mid-SUN subfamilies differentiated by the position of the SUN domain within the protein (Field et al., 2012; Graumann et al., 2014). Furthermore, A Phylogenetic analysis of SUN-domain proteins exhibited an ancient divergence of CCSD (Cter-SUN) and PM3-type (Mid-SUN) protein, and the functional divergence of four orthologous groups (SUN1/2/3/5) within grass species (Murphy et al., 2010). Cter-SUN proteins have been described in *Arabidopsis* (AtSUN1, AtSUN2), maize (ZmSUN1, ZmSUN2), rice (OsSUN1, OsSUN2), yeast (Mps3), *Sordaria macrospora* (SmSUN1), and other organisms (Graumann et al., 2010; Murphy et al., 2010; Friederichs et al., 2012; Vasnier et al., 2014; Varas et al., 2015; Zhang et al., 2020). In yeast, the protein MPS3, critical for vegetative growth and sporulation, is involved in spindle polar body (SPB) replication, spindle formation during mitosis, and fusion of the nucleus (Jaspersen et al., 2002; Nishikawa et al., 2003; Antoniacci et al., 2004). In plants, SUN1 and SUN2 can interact with the KASH-domain of the WIP protein and SINE protein to anchor WIT, forming a NE bridge (also termed LINC complex) (Zhou et al., 2012; Tamura et al., 2013; Graumann et al., 2014; Groves et al., 2020). The LINC complex has multiple functions with structural roles in positioning of nuclei, maintaining the shape of nuclei, movement of the pollen nucleus, stomatal development, and in plant male fertility (Graumann et al., 2014; Tatout et al., 2014; Evans et al., 2020; Groves et al., 2020). Recently, the structure and dynamics research revealed that this bridging complex have a role in seed maturation and germination, Organ development, response to stress, and the regulation of gene activity by organizing chromatin in the 3D nuclear space (Evans et al., 2020). In addition, LINC complexs (SUN1) and lamin-like proteins (CRWN1/4) physically and functionally interact with chromatin-regulatory proteins (PWO1) that play roles in gene expression, nuclear size, and nuclear shape (Mikulski et al., 2019; Groves et al., 2020). A new NE protein, OPENER, was recently identified and binds SUN1/2 and is involved in embryonic development and nucleolar size (Wang et al., 2019). AtSUN1 and AtSUN2 play crucial roles in meiosis (Zhou et al., 2012, 2014). The double mutant of Atsun1-1/Atsun2-2 displayed greatly reduced fertility and severe meiotic defects, such as a delay in the progression of meiosis, an absence of full synapsis, the presence of unresolved interlock-like structures, and a reduction in the mean cell chiasma frequency (Varas et al., 2015). The double mutant of *Ossun1/Ossun2* displayed similar severe defects in meiosis as *Atsun1-1/Atsun2-2*, but *OsSUN2* has a more important role than *OsSUN1* in rice meiosis (Zhang et al., 2020). The maize SUN2 (ZmSUN2) formed a distinct belt-like structure at the nuclear periphery that are converted to a half-belt in zygotene and then back to a belt in pachytene. The half belt structure of ZmSUN2 is disrupted in the chromosome segregation mutants, desynaptic (dy1), asynaptic1 (as1), and divergent spindle1 (dv1) (Murphy et al., 2014). This result suggests that the SUN belt is associated with meiotic telomere dynamics, chromosome synapsis. Mid-SUN proteins, different from Cter-SUN proteins, contain three TM domains (one at the N-terminus and two at the C-terminus), coiled-coil domains, and a Sun domain located in the central area. In yeast, SLP1 protein as the sole Mid-SUN protein is part of the complex with the YERP65 protein recruiting MPS3 localized in the NE (Friederichs et al., 2012). In *S. macrospora*, the deletion mutant of the *SLP1* gene shows defects in both vegetative growth and sporulation (Vasnier et al., 2014). The Mid-SUN proteins have been described in *Arabidopsis* (AtSUN3, AtSUN4, and AtSUN5) and maize (ZmSUN3, ZmSUN4, and ZmSUN5) (Murphy et al., 2010; Graumann et al., 2014). AtSUN3 and AtSUN4 are located in the NE and ER, while ZmSUN3 and ZmSUN4 are located only in the NE (Murphy et al., 2010; Graumann et al., 2014). AtSUN3 and AtSUN4 are expressed in many tissues at moderate levels, while AtSUN5 is mainly expressed in pollen and various embryonic tissues (Graumann et al., 2014). In maize, *ZmSUN3* and *ZmSUN4* share similar expression patterns with those of *AtSUN3* and *AtSUN4* (Murphy et al., 2010). *ZmSUN5* is specifically expressed in pollen, suggesting the function of nuclear migration down the pollen tube and possibly double fertilization (Murphy et al., 2010). In *Arabidopsis*, the single mutants in *Atsun3-1*, *Atsun4-1*, and *Atsun5-1* do not show obvious growth or fertility defects, although changes in nuclear morphology can be detected (Graumann et al., 2014). The mutants of *AtSUN3/Atsun3-1*, *Atsun4-1*, and *Atsun5-1* produced approximately 17.4% of aborted seeds/siliques, and the homozygous triple mutant was lethal (Graumann et al., 2014). In addition, Membrane yeast two-hybrid (MYTH) assay provides evidence for a complex which is formed by interaction of Mid-SUN proteins with Cter-SUN proteins through their coiled-coil domains on the NE, but the biological functions have not been confirmed in previous studies (Graumann et al., 2014). However, while SUN proteins are highly conserved, and exist in most organisms, the evolutionary history of Cter-SUNs and Mid-SUNs has not been systematically studied, and the function of mid-SUN proteins are poorly studied, especially SUN5. In this study, a combination of bioinformatics and molecular experiments were conducted to illuminate the evolution and the divergence of the Cter-SUN proteins and Mid-SUN proteins. Our results showed that the Cter-SUN and Mid-SUN proteins were monophyletic and have undergone different evolutionary histories from protists to plant species. Different expression patterns of the SUN members in cotton indicated the functional divergence among the subfamilies. Decreasing the expression of *GbSUN5* caused the abortion of cotton seeds, indicating a probable function during fertilization, different from *GbSUN3*.

## Materials and Methods

### Data Sources and Sequence Retrieval

To obtain as many as *SUN* genes in sequenced eukaryote genomes as possible, several datasets and multiple steps were used to search for the sequences. Protein sequences of protists and fungi were downloaded from the Ensembl database^1^ ^,2^. Plant proteomics were downloaded from the Phytozome database^3^. The sequences of cotton and *Arabidopsis* were retrieved from CottonFGD^4^ and Tair datasets^5^. We also obtained the prokaryotic protein sequences from the Ensembl Bacteria database^6^. The amino acid sequences of *AtSUN* genes (Graumann et al., 2014) were used as queries for gene searches using BLASTP for *SUN* genes in the several datasets mentioned above within representative species with a cut-off *E*-value set at 1e^–2^. These sequences were further verified via Pfam (El-Gebali et al., 2019) batch searches with default settings for the threshold option, a Conserved Domain Database (CDD) batch search (Marchler-Bauer et al., 2017) and SMART database batch search (Letunic et al., 2015). Sequences with obvious errors and/or lengths less than 150aa were removed manually. Confirmed sequences were used for further analysis.

### Sequence Alignment and Phylogenetic Analyses

The amino acid sequences of SUN proteins were aligned using MUSCLE 3.8.31 (Edgar, 2004) with default parameters. MEGA-X (Kumar et al., 2018) was used to find the best model and to construct the maximum likelihood (ML) tree with bootstrap tests of 1000 replicates, the Gamma Distribution option, the partial deletion option, and the JTT + G model.

### Motif Analyses, Gene Structure, and Prediction of Domain Organization

All SUN protein sequences were used to search against the Pfam (El-Gebali et al., 2019) HMMER (Potter et al., 2018), and CDD (Marchler-Bauer et al., 2017) databases to find other known domains/motifs apart from the SUN domains. To discover novel conserved motifs, the software Multiple Em for Motif Elicitation (MEME) v5.1.1 (Bailey et al., 2009) was employed online^7^ using the following parameters: Zero or One Occurrence Per Sequence (zoops), and the number of motifs was no greater than 20. The Gene Structure Display Server^8^ (Hu et al., 2015) was used for gene structure analysis. TMHMM v2.0^9^ (Krogh et al., 2001) and Tmpred^10^ (Hofmann and Tmbase, 1993) were used to predict transmembrane helices (TMH). Coiled-coil (CC) domains were predicted by COILS-Server^11^ (Lupas et al., 1991).

### Inference of Gene Duplication and Loss Events

The plant species tree was adapted from TimeTree^12^ (Kumar et al., 2017). The gene trees obtained from MEGA-X for each of two SUN clades were reconciled with the plant species tree individually by Notung-DM (Chen et al., 2000; Darby et al., 2017) with default parameters.

### Expression Profiles of *GhSUN* and *GbSUN* Genes

To analyze the expression profiles of *GhSUN* and *GbSUN* genes in different tissues and developmental stages, expression data for mRNA levels were retrieved from the genome-wide RNA-seq dataset in CottonFGD^13^ (Zhu et al., 2017) and the Cotton Omics Database^14^ (Hu et al., 2019), respectively. The heatmap charts were drawn according to gene expression values (FPKM).

### Plant Materials and Growth Conditions

*Arabidopsis* Columbia (Col-0) was used for the *GbSUN5_At* promoter transfer experiments and as wild-type controls. Plants were grown on soil in a growth chamber under long-day conditions (16 h light/8 h dark) at approximately 22–24°C. *Gossypium hirsutum* acc. TM-1 was used for the VIGS assay. TM-1 was grown in pots at 25°C in a growth chamber under a 16 h light–8 h dark cycle with 60% humidity.

### RNA Extraction and Quantitative Real-Time (qRT)-PCR

Total RNA was extracted from different tissues using the RNAprep Pure plant Kit (Tiangen, Beijing, China). RNA was reverse transcribed to cDNA using a PrimeScript^®^ RT reagent kit (Takara, Dalian, China). PCR amplifications were performed using SYBR^®^ Premix Ex Taq^TM^ (Tli RnaseH Plus) on an Applied Biosystems 7500 Fast Real-Time PCR System. The PCR conditions were as follows: primary denaturation at 95°C for 30 s followed by 40 amplification cycles of 5 s at 95°C and 30 s at 60°C. Cotton Actin7 (CottonFGD Gene ID: Gbar_A11G005750) was used as an internal control. Melting curve analysis was performed to ensure there was no primer-dimer formation. Information of the qRT-PCR primers are presented in Supplementary Table 1. The qRT-PCR was carried out with three biological replicates, each comprising three technical replicates. Relative gene expression levels were calculated using the 2^–ΔΔ^^Ct^ method (Livak and Schmittgen, 2001).

### Promoter Activity Analysis and Plant Transformations

To further analyze the expression pattern of *GbSUN5_At*, two *GbSUN5_At* upstream fragments of Hai 7124 (containing 1793 and 3149 bp at upstream locations of the ATG start codon) were amplified with the primer pair Ps1/Ps2 and Ps3/Ps4 (Supplementary Table 1) and recombined into PBI121 (GUS reporter gene) and pcambia2300 [green fluorescent protein (GFP) reporter gene], respectively, by In-Fusion^®^ Cloning. The two constructs were both introduced into the host cells of *Agrobacterium tumefaciens* LBA3101. *Agrobacterium*-mediated transformation of *Arabidopsis thaliana* was transformed using the floral-dip method (Clough and Bent, 1998) and a modified procedure for *Agrobacterium* preparation (Logemann et al., 2006).

### Histochemical Detection of GUS Activity and DAPI Staining

GUS (beta-glucuronidase) histochemical staining was performed using the GUS Staining Kit (Coolaber, Beijing, China). Tissues were stained with GUS solution overnight at 35°C after vacuum infiltration. Pollen was stained with DAPI (4′, 6-diamidino-2-phenylindole, dihydrochloride) as described in Coleman and Goff (1985).

### Analyses of Pollen Viability

The mature pollen from VIGS plants, knockout mutants, and the WT were fixed overnight in Carnoy’s fluid (60% ethanol, 30% chloroform, 10%, glacial acetic acid) and washed in a graded ethanol series (95% [×3], 75% [×3]). Fixed pollen was stained by Alexander’s staining solution for testing viability (Alexander, 1969).

### VIGS Assay

We isolated a 450-bp fragment of *GhSUN5_At* from TM-1. The fragment was amplified using primers Psv5/Psv6 and subcloned into *Spe*I and *Sac*I digested pCLCrVA, generating pCLCrVA-*GhSUN5_At* constructs (Tuttle et al., 2008). The vectors pCLCrVA-*GhSUN5_At* and pCLCrVB were introduced into *A. tumefaciens* strain GV3101 (Idris and Brown, 2004). More than 50 individual plants were infiltrated with a mixture of *A. tumefaciens* carrying pCLCrVA-*GhSUN5_At* and pCLCrVB (Idris and Brown, 2004). Untreated (CK) and empty vector (CLCrv: 00) transformed plants (*n* > 50) were used as experimental controls. The transcript levels of *GhSUN5* in mature pollen of silenced plants were detected using primers Psv7Psv8 (primers indistinguishable between homologs). Those primers were listed in Supplementary Table 1.

### CRISPR/Cas9 Construction and Cotton Transformation

For targeted editing of GhSUN5, a pair of sgRNAs was designed in the coding region of the SUN domain, and the tRNA-sgRNA fragment was ligated to the Prgeb32-GhU6.9-NPT II expression vector. Primers are listed in Supplementary Table 1. The construct was introduced into the host cells of *A. tumefaciens* LBA4404. Cotton cultivar Gh cv. HM-1, which exhibits a normal growth habit, was used as the transformation receptor. *Agrobacterium*-mediated transformation was conducted following a previous report (Jin et al., 2006).

## Results

### Early Divergence of SUN Proteins in Plants

**FIGURE 1:**
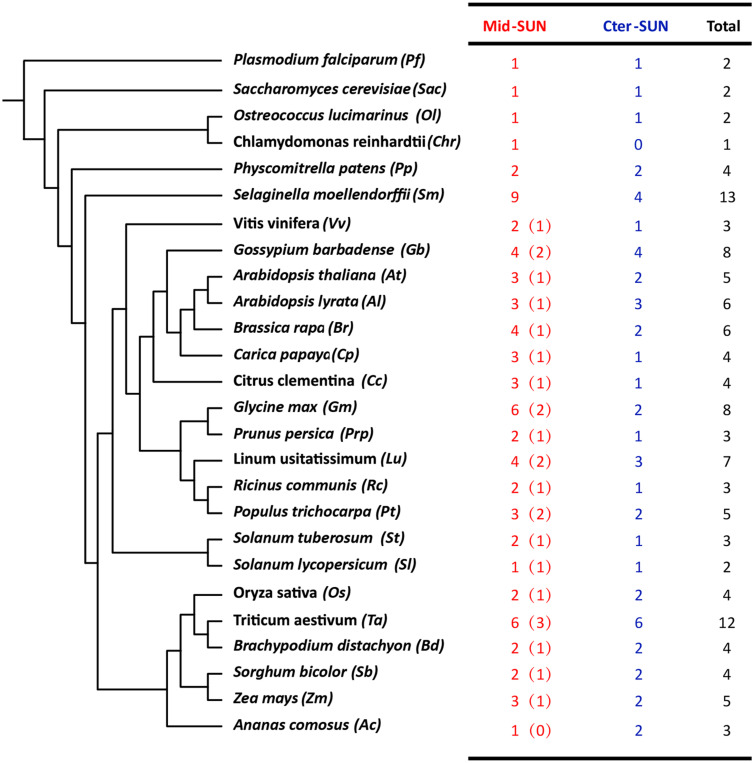
The number and classification of *SUN* genes in representative species. The numbers in the brackets of Mid-SUN denote the number of *SUN5* genes in each Angiosperm species.

**FIGURE 2:**
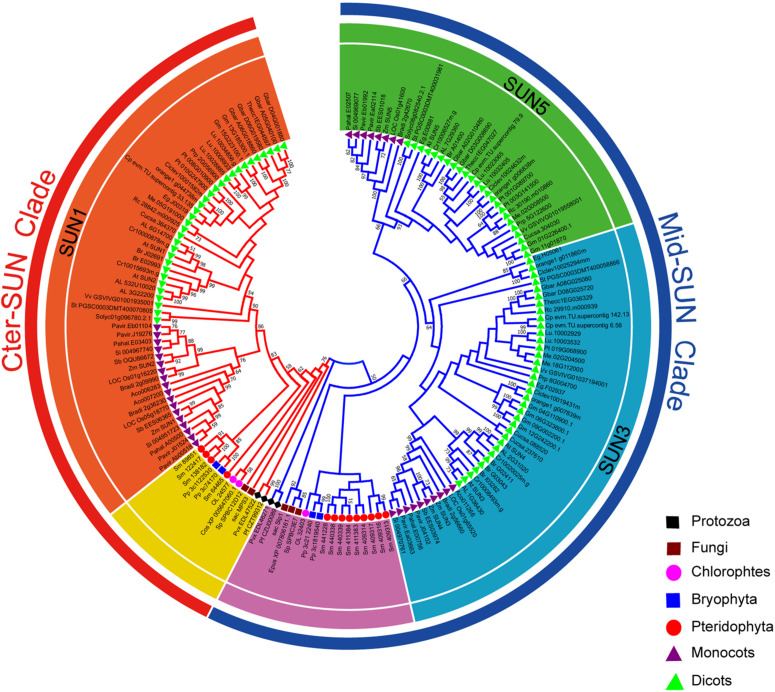
Phylogeny of representative *SUN* genes from protists, plants, and fungi. The tree topology generated via MEGA-X is shown here. Numbers on branches indicate the bootstrap percentage values calculated from 1000 replicates, and only values higher than 50% are shown. The Mid-SUN clade is indicated by the blue branch lines, and the Cter-SUN clade by the red branch lines. The Mid-SUN clade is separated into SUN3 and SUN5. The Cter-SUN clade retained one subfamily of SUN1. The peripheral groups of Cter-SUN and Mid-SUN are shown in yellow and purple, respectively.

In plants, members of the SUN gene family are characterized by three important units, transmembrane (TM), coiled-coil (CC), and SUN domains (Graumann et al., 2010; Murphy et al., 2010; Oda and Fukuda, 2011). To identify the *SUN* genes in major lineages, we performed searches against plants, protists, and fungi using the SUN proteins from *A. thaliana* (Graumann et al., 2014). In all, 216 sequences were retrieved from the genomes of 42 plants, 6 fungi, and 4 protists (Figure 1 and Supplementary Tables 2, 3). To explore the evolutionary history of eukaryotic *SUN* genes, we conducted phylogenetic analyses with full-length sequences from 26 representative species (Figure 1) using MEGA-X. Phylogenetic analysis of the retrieved proteins suggested that SUN proteins in eukaryotes evolved into two monophyletic clades (Cter-SUNs and Mid-SUNs) (Figure 2). In the selected species, the copy number of *SUN* genes ranged from 2 in *Saccharomyces cerevisiae* to 8 in soybeans, with the highest number being 13 in *Selaginella moellendorffii*. Two kinds of *SUN* genes were found in almost all selected species except for *Chlamydomonas reinhardtii* and *Coccomyxa subellipsoidea* C-169 (Supplementary Table 2). Two major clades of SUN genes in Mesangiospermae went through different evolutionary processes. Mid-SUN genes further evolved into two distinct subgroups (termed as *SUN3* and *SUN5*) before the divergence of the ancestor of angiosperms, while Cter-SUNs retained one subfamily of *SUN1* (Figure 2).

To explore the origin of the *SUN* gene family, CLIME^15^ (Li et al., 2014) was performed for predicting the evolutionarily conserved modules (ECM) using At-SUN proteins. The results showed two distinct evolutionary histories between Cter-SUNs and Mid-SUNs across 138 eukaryotes. Mid-SUN proteins existed in more protists and fungi, with first appearance in *Entamoeba histolytica*, while Cter-SUN was lost in many fungi, with the first appearance in *Plasmodium vivax* (Additional File 1). Analysis of the *SUN* genes identified in the 10 species of protists and fungi revealed a similar distribution into two clades (Supplementary Figure 1). This further suggested that the division between these two branches dated from before the divergence of the protists.

### Two Types of SUN Proteins in Angiosperms

**FIGURE 3:**
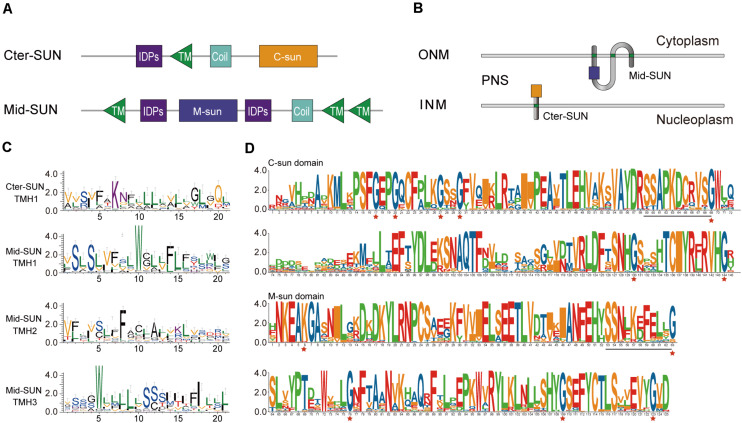
Protein organization and sequence features of SUN proteins. **(A)** Comparative protein motifs of the Cter-SUN and Mid-SUN protein subfamilies. Protein motifs are drawn based on a search using CDD, Pfam, HMMER, and MEME programs. **(B)** Possible protein arrangement models with the SUN domain in the perinuclear space are shown for the Cter-SUN and Mid-SUN. Models do not attempt to depict other domain organizations (such as IDPS and coiled-coil) and multimer interactions that may occur with the SUN or coiled-coil (not shown) domains. PNS, perinuclear space at the nuclear envelope. **(C)** Sequence features shown in the form of web logos representing TMH of two clades of Cter-SUNs and Mid-SUNs from 26 selected species. Logos were generated using the Weblogo3 application (http://weblogo.threeplusone.com/). **(D)** SUN domain-logo analysis of Cter-SUN and Mid-SUN proteins in 20 angiosperm species. The red stars indicate conserved Gly motifs in two types of SUN proteins. Common conserved motifs of C-SUN domain and M-SUN domain are underlined.

According to the Pfam database at http://pfam.sanger.ac.uk, the SUN-domain protein family comprises over 30 different architectures that can be grouped into proteins with a central SUN domain (Mid-SUNs) and proteins containing a SUN domain at their C-terminus. Based on a combination of type and number of the TMH motif(s), coiled-coil (CC) (s), intrinsically disordered protein regions (IDPs), and the Sad1/UNC-84 domain, all of the SUN proteins from angiosperms can be further classified into two types (Cter-SUNs and Mid-SUNs). The Cter-SUN proteins usually contain a SUN domain at the C-terminus (C-sun) and a TMH motif at the N-terminus with CC and IDPs. The Mid-SUN proteins contain three TMH motifs (one TMH motif at the N-terminus and two TMH motifs at the C-terminus) and one other type of SUN domain (M-sun) with an internal CC and IDPs (Figure 3A and Supplementary Figure 2). The TMH in Cter-SUN showed moderately conserved amino acid residues. *AtSUN1* and *AtSUN2* were located in the INM of the NE, showing the transmembrane domain from nucleoplasm to the NE lumen (Zhou et al., 2012). AtSUN3 and AtSUN4 proteins expressed as fluorescent fusion proteins were membrane-associated and localized to the NE and ER (Graumann et al., 2014). The sequence logos of TMHs from two clades of proteins showed two types of TMH units. TMH1 of Cter-SUN and TMH2 of Mid-SUN shared similar amino acid residues enriched with Val, Ser, Phe, and Leu (Figure 3C), suggesting the similar directions from nucleoplasm to cytoplasm. TMH1 and TMH3 of Mid-SUN contained conserved Trp residues and moderately conserved Ser and Leu (Figure 3C), showing the same direction from cytoplasm to the lumen of the NE. Thus, the model of topological arrangements for generalized Cter-SUN and Mid-SUN proteins in the plants NE are presented in Figure 3B. Examination of the functional units of two subfamily members (SUN3 and SUN5) of Mid-SUN revealed little difference in angiosperms, except for the CC units. In *S. moellendorffii*, *Physcomitrella patens*, and *Ostreococcus lucimarinus*, none or partial TMHs existed in the proteins of Mid-SUN (Supplementary Figure 2).

To better understand the difference between the two types of SUN proteins, exon/intron organization of different *SUN* genes was also examined. Each monophyletic clade shared similar exon/intron organization. Most members of Cter-SUNs usually contained one intron, while those in Mid-SUNs had multiple exons. It was intriguing to note that the SUN domain was separated by an intron in Cter-SUN, but the domain in Mid-SUN was maintained complete without an intron insertion (Supplementary Figure 3).

### Each Monophyletic Clade Defines One Type of Sad1/UNC-84 Domain in Plant SUN Proteins

To further examine the divergence between the two types of Sad1/UNC-84 domains in SUN proteins, the conserved protein domains from 20 selected angiosperm species were filtered out. We performed sequence logo analysis for 68 SUN domain sequences from Mid-SUNs and 40 from Cter-SUNs. Examination of the domains revealed that their protein sequences shared unique characteristics within each of the two SUN phylogenetic clades (Figure 3D). The Cter-SUN proteins (SUN1) share the same type of Sad1/UNC-84 domain (hereby named the “C-sun” domain), which was also displayed by conserved motifs (motifs 4, 7, 11, and 13) (Supplementary Figure 2). Mid-SUN proteins (SUN3 and SUN5) shared another type of Sad1/UNC-84 domain (hereby named the “M-sun” domain), which was also displayed by conserved motifs (motifs 1, 2, 5, and 6) (Supplementary Figure 2). The C-sun domain contains a consensus conserved seven-Gly motif, while the M-sun domain contains highly conserved five-Gly motifs (Figure 3D). The C-sun domain starts with conserved Pro-Ser-Phe-Gly-Glu-Pro-Gly, ends with Thr-Cys-Iie-Tyr-Arg-X-Arg-Val-His-Gly, and has stretches of about 20 amino acid residues with lower conservation in the middle. The M-sun domain started with conserved Asn-Lys-Glu-Ala-Lys-Gly-Ala, ended with Cys-Thr-Leu-Ser-X-X-Glu-Val-Tyr-Gly and was more conserved than the C-sun domain. The Sad1/UNC-84 domains between SUN3 and SUN5 from selected angiosperm species shared consensus conserved amino acid residues. However, examination of both types of Sad1/UNC-84 domains in the identified SUN proteins revealed that they shared no consensus conserved motif except for the common SSxxKxxxxxG motif (Figure 3D), suggesting a significant difference of biological function in the two monophyletic clades, but they might have a common ancestor.

### Duplication and Loss of SUN Genes in Plants During Evolution

**FIGURE 4:**
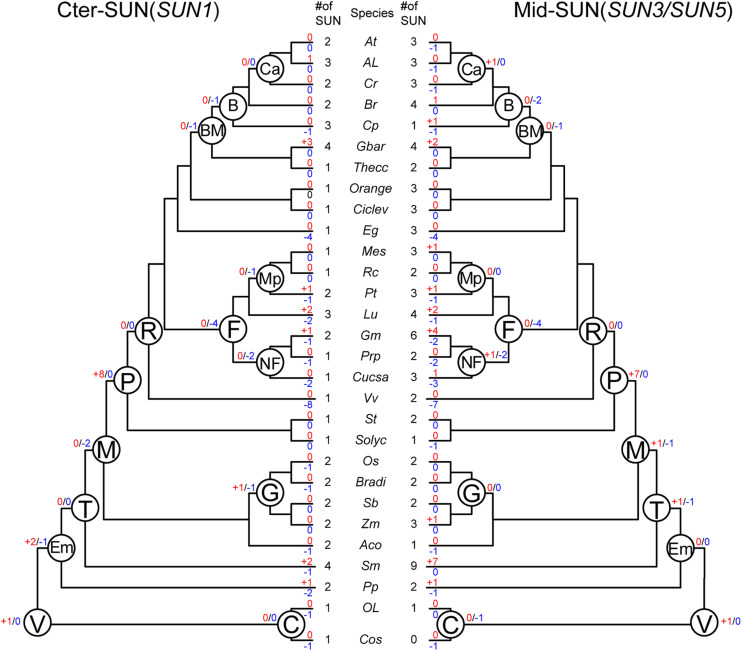
Evolutionary events in the *SUN* gene family in plants. Numbers of gene duplication (shown in blue after “+”) and loss (shown in red after “–”) events were inferred for each internal node as well as for current extant species. The numbers of *SUN* genes are also listed accordingly. *Cos*, *Chlorella vulgaris*. *Ol*, *Ostreococcus lucimarinus*. *Pp*, *Physcomitrella patents*. *Sm*, *Selaginella moellendorffii*. *Aco*, *Ananas comosus*. *Zm*, *Zea mays*. *Sb*, *Sorghum bicolor*. *Bradi*, *Brachypodium distachyon*. *Os*, *Oryza sativa*. *Solyc*, *Solanum lycopersicum*. *St*, *Solanum tuberosum*. *Vv*, *Vitis vinifera*. *Cucsa*, *Cucumis sativus*. *Prp*, *Prunus persica*. *Gm*, *Glycine max*. *Lu*, *Linum usitatissimum*. *Pt*, *Populus trichocarpa*. *Rc*, *Ricinus communis*. *Me*, *Manihot esculenta*. *Eg*, *Eucalyptus grandis*. *Ciclev*, *Citrus clementina*. *Orange*, *Citrus sinensis*. *Thecc*, *Theobroma cacao*. *Gbar, Gossypium barbadense*. *Cp*, *Carica papaya*. *Br*, *Brassicales*. *Cr*, *Capsella rubella*. *Al, Arabidopsis lyrata*. *At*, *Arabidopsis thaliana*. C, Chlorophyta. V, Viridiplantae. Em, Embryophyta. T, Tracheophyta. M, Mesangiospermae. P, Pentapetalae. R, Rosis. F, Fabid. NF, Nitrogen fixing. Mp, Malpighiales. BM, Brassicales Malvales. B, Brassicales. Ca, Camelineae. G, Grass.

To better understand the evolutionary events that have occurred among these two subfamilies, we performed an analysis of gene duplication and loss using Notung software (Chen et al., 2000; Darby et al., 2017). We obtained the number of variations of *SUN* genes at different stages of evolution according to the constructed phylogenies and inferred whether the internal nodes within the each clade were associated with gene duplication, gene loss, or lineage divergence events (Figure 4).

In Vridiplantae, gene duplication was detected, and no gene loss was found in either SUN clade. Cter-SUNs did not experience gene duplication/loss events; Mid-SUN experienced one gene loss event in Chlorophyta. During the emergence of the embryophytes, no gene duplication/loss events occurred in Mid-SUNs, but two *SUN* genes were duplicated and one gene was lost in Cter-SUNs. In tracheophytes, Mid-SUNs experienced one gene loss and one gene duplication. However, during the emergence of Mesangiospermae, two genes were lost and none were duplicated in Cter-SUNs, while one gene was duplicated and lost in Mid-SUNs. These results suggest that Mid-SUN experienced rapid birth-and-death events (Karlin and Mcgregor, 1957; Nam et al., 2004) that may have resulted in the divergence between SUN3 and SUN5. In the grass lineage, Cter-SUNs exhibited one gene loss and one gene duplication; there were no gene duplication/loss events occurring in Mid-SUNs. With the emergence of Rosids, eight and seven genes were duplicated in Cter-SUNs and Mid-SUNs, respectively, and no gene was lost in either clade. In the Fabids, four genes were lost, while no gene was duplicated in either clade. With the emergence of the nitrogen-fixing plants, two genes were lost in Cter-SUNs and Mid-SUNs, but one gene was duplicated in the Mid-SUNs. The Brassicales, Malvales, and Brassicales lost several genes, and no duplicated genes were detected. Only Camelineae had no losses in either Cter-SUNs or Mid-SUNs, but only one duplicated gene was detected in Mid-SUNs (Figure 4).

Furthermore, we also examined gene-duplication/loss events in extant plant species. The gene duplication events in several extant plant species, such as *Glycine max*, were probably associated with their recent whole-genome duplication events. In contrast, in several other plant species including *Vitis vinifera* and *Eucalyptus grandis*, the phylogenies of SUN proteins showed drastic gene loss events. As for the general results, Mid-SUN experienced approximately equal numbers of gene loss events as Cter-SUN, while there were more gene duplications in Mid-SUN than in Cter-SUN (Supplementary Table 4). This observation suggests that more gene duplications in Mid-SUNs may be also a reason for the divergences between SUN3 and SUN5.

### Members of Each SUN Subfamily Share Similar Expression Patterns in Cotton

**FIGURE 5:**
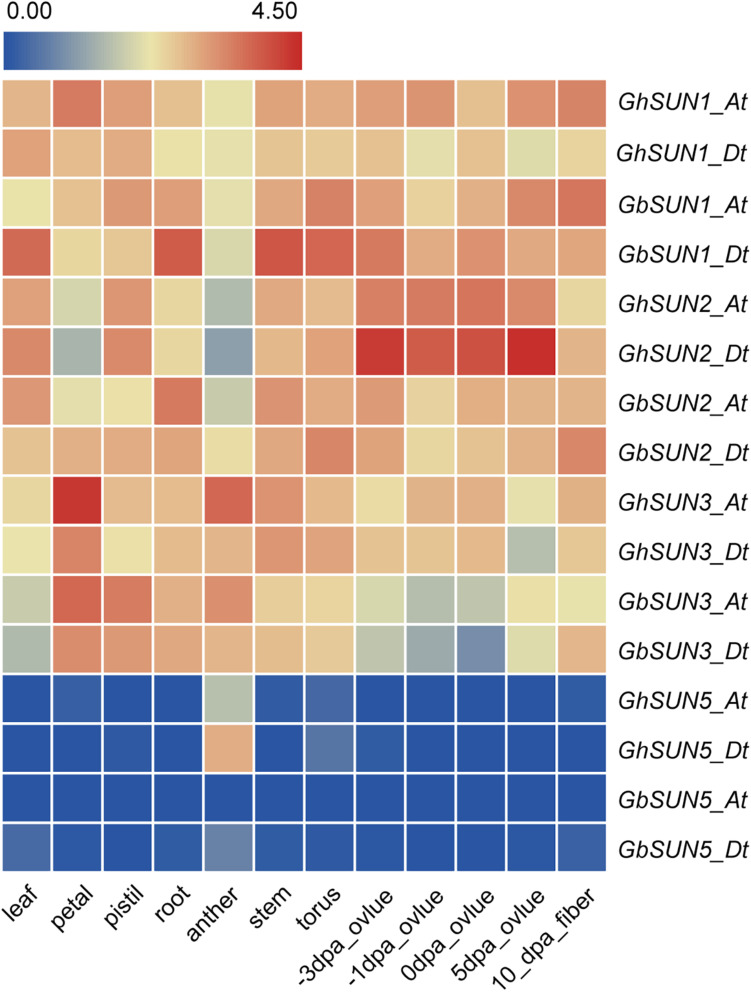
The expression profiles of *SUN* genes in a variety tissues of sea island cotton and upland cotton. The raw data for RNA-Seq of upland cotton and sea island cotton were downloaded from CottonFGD (https://cottonfgd.org/) and Cotton Omics (http://cotton.zju.edu.cn/), respectively. Gene expression levels are depicted with different colors on the scale. Red and blue represent high and low expression levels, respectively. dpa represents day post-anthesis.

To test the hypothesis that Cter-SUN and Mid-SUN evolved independently and to examine the divergence of SUN3 and SUN5, we used RNA-seq data of island cotton (Hai-7124) and upland cotton (TM-1) to analyze the expression patterns of the *SUN* genes in different tissues. Members of *SUN* genes showed a similar expression pattern across various tissues in island cotton and upland cotton, suggesting similar function in regulating plant growth and development. *SUN1s* and *SUN2s* in cotton were expressed at medium levels in detected tissues. *SUN3s* are mainly expressed in petals and stamens and are moderately expressed in other tissues. Interestingly, *SUN5s* are specifically expressed in pollen (Figure 5). These results agree with the similar expression of those in maize and *Arabidopsis* (Murphy et al., 2010; Graumann et al., 2014).

**FIGURE 6:**
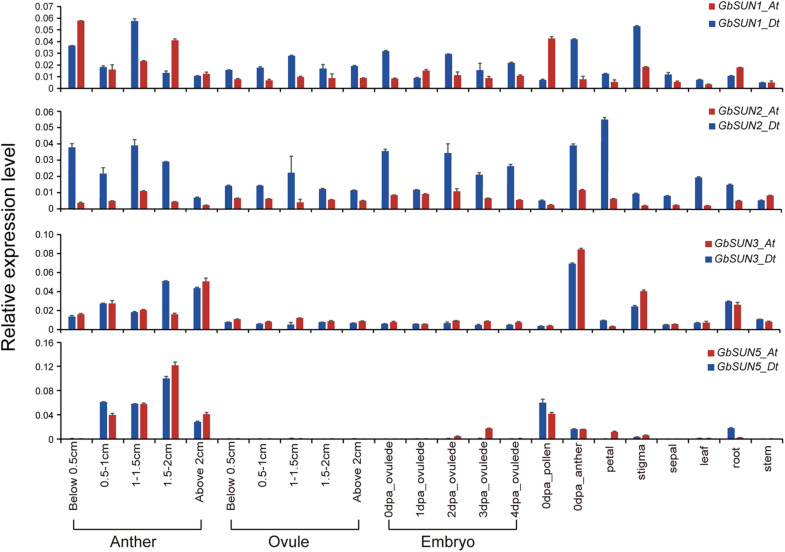
qRT-PCR confirmation of the expression levels of *SUN* genes in G. barbadense. Error bars represent the standard deviations of three independent experiments. Anthers and ovules at different stages were gathered with reference to the length of flower buds. dpa represents days post-anthesis. Values are means of at least three biological replicates.

For verification of the data from RNA-seq, quantitative RT-PCR was used to profile the expression levels of *GbSUN1*, *GbSUN3*, and *GbSUN5* in different tissues of Hai-7124. Cter-SUNs (*GbSUN1* and *GbSUN2*) were found to be ubiquitously expressed, in agreement with published RNA-seq data. The expression levels of Mid-Sun genes in island cotton were clearly distinguished into two groups. Moderate expression of *GbSUN3* was detected at different stages of anther development and in stigmas and roots, while there was lower expression in the other tissues. *GbSUN5* showed specific expression during stamen development and pollen maturation. It was interesting that *GbSUN3* showed expression patterns similar to *GbSUN5* in anthers but expressed differently in the pollen on the flowering day (Figure 6).

The expression level of *GbSUN3* gradually increased with anther development but had the lowest expression in the pollen on the flowering day. However, *GbSUN5* had higher expression in the pollen on the flowering day than in post-dehiscence anthers, indicating a more important core role in pollen development, not in anthers (Figure 6). The different expression patterns among the three subgroups in island cotton suggested the functional divergence between SUN1, SUN3, and SUN5.

### *GbSUN5* Specific in Mature Pollen

**FIGURE 7:**
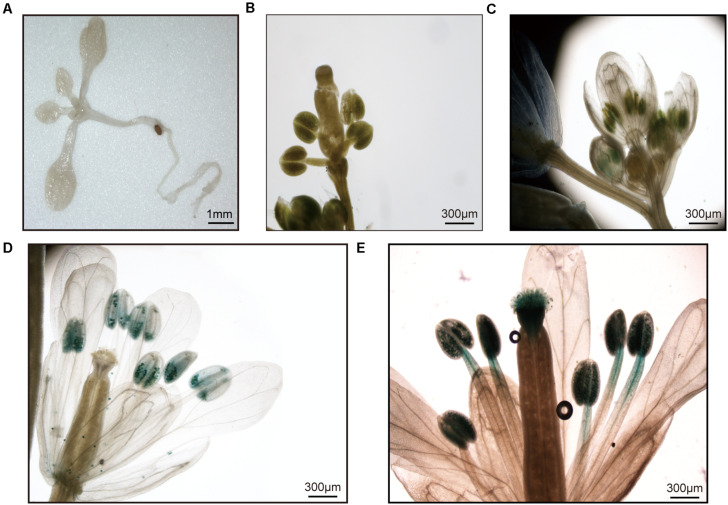
Analysis of *GbSUN5*_At promoter activity in different tissues. **(A,B)** No GUS staining was observed in the young rosettes or young flowers. **(C)** In immature flowers, a weak GUS signal was observed in stamens. **(D)** In mature anthers of the plant, strong GUS staining was observed in mature pollen grains, whereas no GUS staining was observed in the anthers. **(E)** GUS staining was also detected in mature pollen and stigmas several hours after anthesis.

The expression profile of *GbSUN5* restricted to the mature pollen was similar to that in maize (Murphy et al., 2010). To investigate whether *GbSUN5* is specifically expressed in pollen, a 1.8-kb upstream fragment from *GbSUN5_At* and a 1.8-kb upstream fragment from *AtSUN5* were transcriptionally fused to the GUS reporter gene and transformed into *Arabidopsis* Col-0. The results showed that no GUS staining was found in seedlings or early flowers (Figures 7A,B), while a weak GUS signal was detected in the stamens of immature flowers (Figure 7C). However, strong GUS staining was detected in mature pollen, whereas this was absent in anther tissues (Figure 7D). GUS staining was also detected in mature pollen and stigmas when the male flowers blossomed and the pollen dispersed (Figure 7E).

**FIGURE 8:**
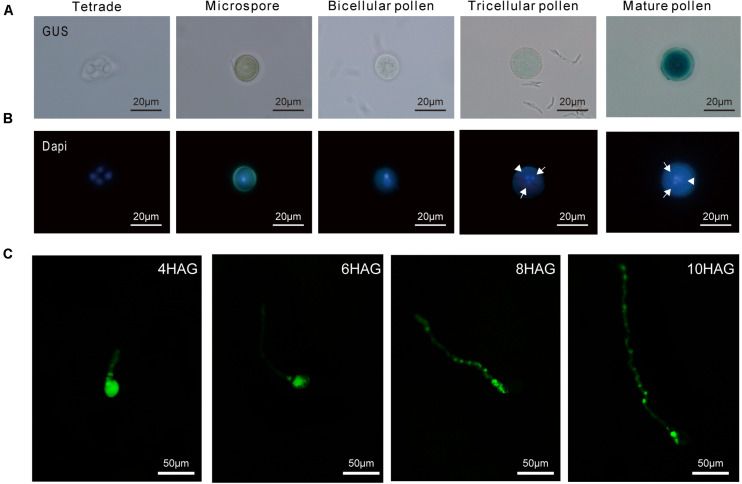
Temporal analysis of *GbSUN5*_At promoter activity in different stages of pollen development. **(A)** GUS staining in different stages of pollen development. **(B)** Fluorescence channels of the same pictures as in panel **(A)**. Determination of pollen stages by visualization of nuclei by DAPI (4′,6-diamidino-2-phenylindole) fluorescence. **(A)** Bright field of the same pictures as in panel **(B)**, showing GUS straining only in pollen grains at the tricellular stage when the sperm cells (white arrow) and vegetative cells (white arrowhead) are present. **(C)** The signals of Pro*GbSUN5*_At-GFP in different stages of pollen germination. After 4–6 h of pollen germination, Pro*GbSUN5*_At-GFP signals were observed in pollen grains and pollen tubes. After 8–10 h of pollen germination, Pro*GbSUN5*_At-GFP signals were found in pollen tubes, whereas no GFP signal was observed in pollen grains. HAG, hours after germination.

To confirm the exact stage, a time course detection during pollen development was performed in GUS transgenic plants. The onset of *SUN5* promoter activity coincided with the second pollen division that leads to tricellular pollen with sperm cells (Figures 8A,B). Similar results were observed in T_1_ lines with Pro*AtSUN5*: GUS (Supplementary Figure 4). The results showed that the specific expression of *SUN5* in mature pollen may be universal in plants, indicating a conservative biological function.

To test whether *GbSUN5_At* was expressed in the grain germination, fusion of GFP with a 3-kb upstream fragment from *GbSUN5_At* transcriptionally was introduced into *Arabidopsis*. Expression of *GbSUN5–GFP* was investigated using fluorescence microscopy during pollen tube germination. The results showed that a strong GFP signal was observed in pollen grains, while a weak signal was detected in pollen tubes 4 h after pollen germination (HAP). At 6 HAP, the GFP signal weakened in pollen grains while being enhanced in pollen tubes. Interestingly, we could still observe a very bright fluorescent signal at 10 HAP (Figure 8C). These results suggested that the promoter of *GbSUN5_At* was active during the entire period of pollen development and germination.

### Knockout and Silencing of *GhSUN5* Resulted in Seed Abortion

To confirm the function of *SUN5*, we isolated a 450 bp fragment from upland cotton (TM1) and inserted it into the VIGS vector (CLCrv) to inhibit the endogenous expression of *GhSUN5* by VIGS. *GhCHLI* (Mg-CHELATASE subunit I)-silenced plants showed a yellow bleaching phenotype as a control to judge whether the expression of *GhSUN5* was silenced successfully in leaves. In Clcrv-*GhSUN5* plants, qRT-PCR analysis revealed that the transcription levels of 22 of 50 VIGS plants were reduced to about 65% (Supplementary Figure 5A), while the pollen viability was similar to that of the CLCrv: 00 plants (Supplementary Figure 5B). At 10 days post-anthesis, abnormal cotton bolls were observed in *GhSUN5*-silenced plants, while the clcrv: 00 plants displayed the wild type (Supplementary Figure 5C). Moreover, some aborted seeds were found in the abnormal bolls (Supplementary Figure 5D). Compared with the Clcrv: 00 plants (approximate abortion rate 3.12%), Clcrv: *GhSUN5* plants had 17.14% aborted seeds (Supplementary Figure 5E).

**FIGURE 9:**
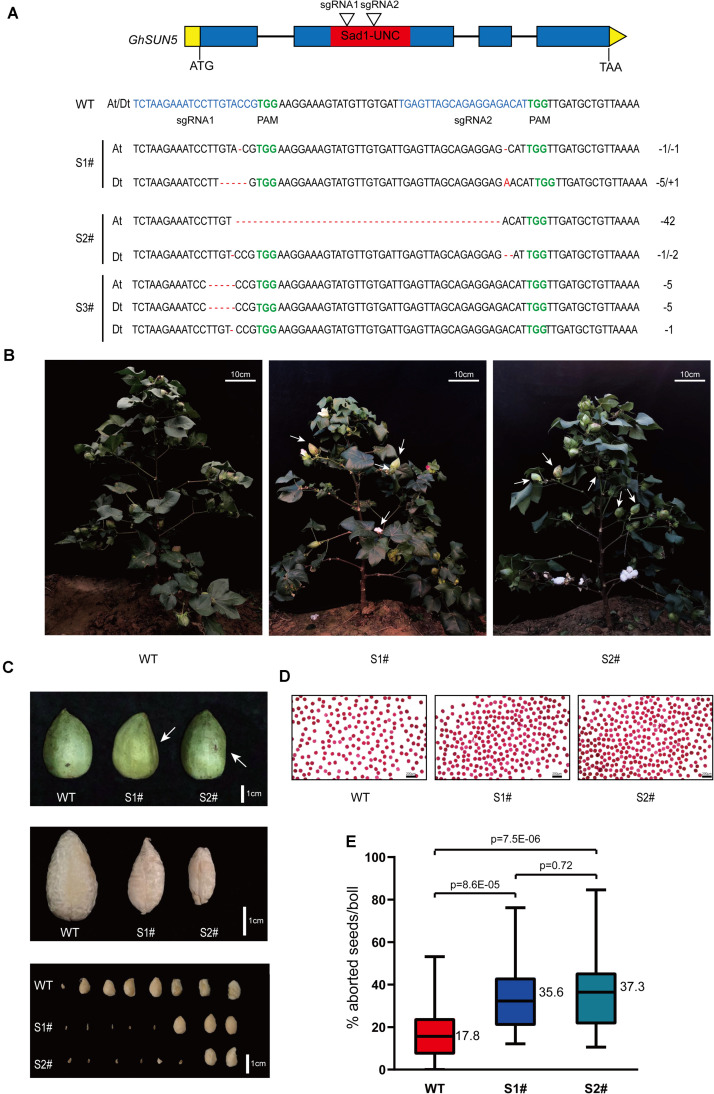
CRISPR-Cas9 targeted editing of the SUN domain in *GhSUN5* genes. **(A)** Schematic map of two sgRNA target sites and three knockout cotton lines (two homozygous and one biallelic mutation). Exons, introns, SUN domain, and untranslated regions are shown as blue blocks, black lines, red blocks, and yellow boxes, respectively. Mutation sites in *GhSUN5* are indicated in red. **(B,C)** Comparison of phenotypes of WT, S1#, and S2# mutants in the boll setting period. White arrows point to abortive cotton bolls **(B)** or aborted seeds **(C)**. **(D)** Pollen vitality assays of S1# and S2# mutants. **(E)** Percentage of aborted seeds per boll in the wild type and the gene-edited plants. The data are shown as box-plot graphs; the horizontal lines across range bars represent the median (*n* > 30) and statistical analysis results from Student’s *t*-test.

To further assess the function of *GhSUN5*, we used the CRISPR-Cas9 system to edit the sequence of the SUN domain against two target sites. Twelve independent transgenic lines were obtained. Three *GhSUN5* knockout lines (two homozygous and one biallelic mutation) with either insertions or deletions at the SUN domain were identified (Figure 9A). Those knockout lines display no obvious growth and pollen abortion (Figures 9B,D). This is similar to the phenotype of VIGS Plants but with more extensive abortion in cotton bolls (Figures 9B,C,E), possibly due to more completely silence of the target gene expression by CRISPR-Cas9 system. These results further suggested that *GhSUN5* genes did not have effects on pollen viability, and that they play a novel role in the karyogamy, sperm or/and early embryo development, similar to the *SLP1* gene in *S. macrospora* and the SUN5 gene in mice and *A. thaliana* (Graumann et al., 2014; Vasnier et al., 2014; Shang et al., 2017).

## Discussion

### Differential Evolution of Cter-SUNs/Mid-SUNs With Conservative and Divergent Patterns in Angiosperms

SUN family proteins are highly conserved but partially differentiated throughout evolution in plant systems (Graumann et al., 2014; Tatout et al., 2014). In most organisms, the copy number of SUN genes varies slightly from ranged from 2 to 8 (Supplementary Table 2). Interestingly, we found that *S. moellendorffii* has the highest number of SUN genes (four Cter-SUN, nine Mid-SUN), which is significantly more than other species (Supplementary Table 2). A recent study showed that the Embryophyta possess at least two ONM KASH proteins (SINEs, WIPs, or TIK) except for *S. moellendorffii*, which interact with INM SUN proteins forming a NE bridge (LINC complexe) (Poulet et al., 2017). In addition, INM Cter-SUNs interacts with mid-SUN proteins forming LINC complexe by CC domain (Graumann et al., 2014). Thus, more Cter-SUNs and Mid-SUNs are required for LINC complexe in *S. moellendorffii* lacking ONM KASH proteins. In early eukaryotes such as fungi, protists, and Chlorophyta, *SUN* genes are present in single or low copy numbers and are essential for viability (Supplementary Table 2). For example, Cter-SUN proteins characterized in *Schizosaccharomyces pombe* and *Caenorhabditis elegans* are involved in duplication of the SPB, nuclear migration, and meiotic chromosome movements (Hagan and Yanagida, 1995; Malone et al., 1999). Following gene duplication, Cter-SUNs developed into SUN1 and SUN2, and these have been found to play vital roles in chromosome movements. In *Arabidopsis* and *Oryza sativa*, loss of *SUN1* and *SUN2* leads to a delay in the progression of meiosis, absence of full synapsis, defects in telomere clustering, and a reduction in the mean cell chiasma frequency. In addition, the expression pattern of Cter-SUN was similar between *Arabidopsis* and maize; *SUN1* and *SUN2* have been shown to be widely expressed in various tissues (Murphy et al., 2010; Graumann et al., 2014; Zhang et al., 2020). Consistent with this, the quantitative RT-PCR results showed that Sea island cotton *SUN1* and *SUN2* were expressed at low levels in most tissues examined in this study (Figure 6). This result indicates that *SUN1* and *SUN2* have maintained relatively conserved functions in meiosis from monocot to dicot plants. However, the functions of SUN1 and SUN2 proteins have diverged during evolution in some angiosperms. In *Arabidopsis*, *AtSUN1* and *AtSUN2* are thought to have completely redundant functions during meiosis. Homozygous single mutants exhibited normal vegetative growth, no obvious loss of fertility, and normal meiotic progression. The double mutant of *sun1* and *sun2* showed a significant reduction in fertility and severe meiotic defects (Varas et al., 2015). In contrast, *OsSUN2* plays a more critical role than *OsSUN1* in rice meiosis. The *Ossun1* single mutant had a normal phenotype, but meiosis was disrupted in the *Ossun2* mutant. These results are consistent with phylogenetic analyses of SUN1 and SUN2 in rice and *Arabidopsis*. *AtSUN1* and *AtSUN2* were closely related to each other, whereas *OsSUN1* and *OsSUN2* were assigned to two separated clades in phylogenetic analyses (Zhang et al., 2020). Interestingly, our phylogenetic analysis found that most monocotyledon SUN1 and SUN2 are divided into two separate clades, but almost all dicotyledon SUN1 and SUN2 were closely related to each other (Figure 2). This result suggested that Cter-SUNs (SUN1 and SUN2) show differential evolutionary patterns between dicotyledons and monocotyledons: Compared with dicotyledons, functional divergence may be more significant in monocotyledons.

Similarly, in the Cter-SUN clade, Mid-SUN also showed conservative and divergent evolution patterns in higher plants. Based on our phylogenetic study, Mid-SUN clade proteins were further evolved into SUN3 and SUN5 subfamilies. Previous studies have demonstrated that *SUN3s* are expressed at low to medium levels in most tissues, whereas *SUN5s* showed a very distinct and much more restricted pollen-related pattern of expression in *Arabidopsis* and *Zea mays* (Murphy et al., 2010; Graumann et al., 2014). This is consistent with expression profiles *GbSUN3-At/Dt* and *GbSUN5-At/Dt* (Figures 6–8). These results imply that SUN3 and SUN5 retained rather conserved functions in higher plants. However, some angiosperm SUN3 subfamily developed into SUN3 and SUN4 by duplication, but it is difficult to distinguish SUN3 from SUN4. In *Arabidopsis*, *AtSUN3*, *AtSUN4*, and *AtSUN5* are thought to have redundant functions during growth and development; the single mutants display no obvious loss of growth or fertility defects, but the triple mutant *sun3 sun4 sun5* was lethal, and in *SUN3/sun3-1 sun4-1 sun5-1* plants, a slight reduction (about 17%) in fertility was observed (Graumann et al., 2014). Unlike *Arabidopsis*, when decreasing the expression of *GbSUN5* by VIGS and disrupting *GbSUN5* by CRISPR/Cas9 systems, aborted seeds were observed in silenced plants (Figure 9). These results suggest the functional divergence of *GbSUN3* and *GbSUN5*. Consistent with this, *GbSUN3* is mainly expressed in anthers instead of pollen, and *GbSUN5* is specifically expressed in pollen (Figures 6–8). In addition, the cotton SUN3 subfamily did not develop SUN3 or SUN4, and Mid-SUN only contains SUN3 and SUN5. These findings led us to propose that there is functional divergence of *GbSUN3* and *GbSUN5* during double fertilization and embryo development, which is quite different from the lack of divergence of their *Arabidopsis* counterparts. At the same time, we noted that some of the SUN3 subfamilies in angiosperms developed into SUN3 and SUN4 by duplication, while the other SUN3 subfamilies did not replicate or lose SUN3 or SUN5, for example, *Ananas comosus* and *Solanum lycopersicum*. These data suggest that the functions of Mid-SUN proteins have had varied patterns of differentiation during the evolution of angiosperms.

### A Model for the Evolutionary History of the *SUN* Gene Family

Our results indicated that Cter-SUN had strikingly different patterns of evolution from Mid-SUN, but both types of SUN-domain proteins may have the same origin. All SUN amino acid sequences were used to search the Pfam databases, and we found that two types of SUN domain belonged to the same Pfam (Pf07738). In addition, although sequence features revealed a significant degree of divergence between c-sun and m-sun in plants through mass evolutionary events, we still found that they have a common SSxxKxxxxxG motif (Figure 3D). This indicates that Cter-SUN and Mid-SUN have a common ancestor and that they evolved independently. In prokaryotes, a few SUN proteins have been identified; these exist as single SUN domains. Individual strains of bacteria contain one gene encoding a SUN domain (Pf07738) (Supplementary Table 5). This observation suggests a role for the SUN domain in fundamental biological processes that have been conserved since prokaryotes throughout evolution. To explore the evolutionary history of prokaryote *SUN* genes, we conducted phylogenetic analyses with SUN domain sequences from prokaryotes and *Arabidopsis*. Based on our phylogenetic analyses, the prokaryote *SUN* genes also can be divided into two monophyletic clades (Cter-Sad1-UNC and Mid-Sad1-UNC) (Supplementary Figure 6). However, there is a single SUN type (Cter-SUN or Mid-SUN) in each prokaryote species. Thus, Cter-SUN and Mid-SUN have some overlapping functions, and absence of one of these types is not critical for survival.

**FIGURE 10:**
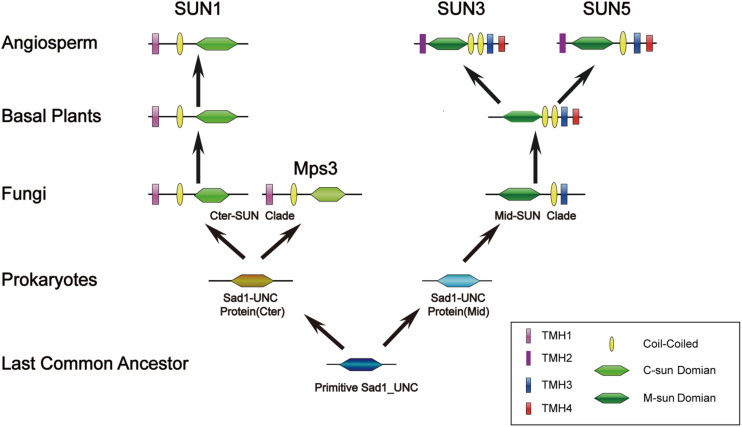
Evolutionary scenario of the *SUN* gene family in plants. The primitive Sad1_UNC (SUN) protein only exists as a SUN domain and may have emerged in the most recent common ancestor. The *SUN* genes were further duplicated and gradually evolved into two clades (Cter-SUN and Mid-SUN) in prokaryotes. Cter-SUN gained an N-terminal TMH and a CC domain, whereas Mid-SUN gained a C-terminal TMH and a CC domain in picoeukaryotes. In yeast, Cter-SUN evolved into Mps3, whereas Cter-SUN developed into SUN1 in other eukaryotes. Through the evolutionary divergence of Angiosperm plants, Mid-SUN proteins gained a C-terminal TMH and gradually evolved into SUN3 and SUN5.

Unlike prokaryote SUNs existing as a single SUN domain, most eukaryote SUN proteins also contain a TMH domain and a coiled-coil domain, and each type of SUN protein (Cter-SUN and Mid-SUN) is highly conserved, especially in the SUN domain (Supplementary Figures 1, 2). However, the homologs of Cter-SUNs MPS3 in *S. cerevisiae* have diverged significantly from the rest of the eukaryotes (Supplementary Figure 7). A blast search using most of Cter-SUN identified the *S. pombe* SUN domain protein with a significant *E*-value but not the Mps3 domain of *S. cerevisiae*. We discovered that Mps3 existed in a few yeast species and was well-conserved (Supplementary Figure 8). At the same time, ECM of yeast SLP1 (Mid-SUN) and MPS3 (Cter-SUN) were predicted by CLIME (Additional File 2). SLP1 homologs are widely distributed in 138 species, and this pattern coincides with *AtSUN3*/*AtSUN4*/*AtSUN5*. In contrast to *AtSUN1*/*AtSUN2*, MPS3 homologs exist only in a few yeasts, in agreement with the above results. It is noteworthy that *AtSUN1*/*AtSUN2* homologs were absent in a few yeasts, while MPS3 homologs were present in those yeasts (Additional File 1). The results indicate that Cter-SUN likely experienced the divergence between yeasts and other eukaryotes. Also, SUN-KASH bridges span the nuclear periplasm and link the nucleoskeleton and cytoskeleton in most organisms, while MPS3 cannot bind to a KASH domain-containing protein due to the lack of many of the residues that are thought to be critical for SUN-KASH binding based on crystallographic studies (Friederichs et al., 2012). However, MPS3 directly or indirectly interacts with some SPB-related proteins such as MPS2, SPC42. NDJ1, SIR4, SPC29, and CSM4, proteins that are yeast-specific (Additional File 3) and are involved in telomere tethering, gene inactivation, formation of the chromosome bouquet, rapid telomere movement in meiotic prophase, SPB duplication, and tethering the half-bridge to the core SPB in *S. cerevisiae* (Jaspersen et al., 2002, 2006; Bupp et al., 2007; Conrad et al., 2008). The function of *S. cerevisiae* MPS3 is similar to other Cter-SUNs (Ding et al., 2007; Talamas and Hetzer, 2011; Friederichs et al., 2012). This finding suggests that a basic mechanism of Cter-SUN protein action is conserved in all eukaryotes but involves a different protein in yeast. In plants, Cter-SUN genes are highly conserved and have retained one subfamily of SUN1, but Mid-SUN genes further evolved into two distinct subgroups (SUN3 and SUN5) before the divergence of the ancestor of angiosperms (Figure 2). Based on the results, we propose a model to describe the evolutionary history of the *SUN* gene family (Figure 10). In this model, we suggest that a primitive SUN protein existed by itself as a single SUN domain in the common ancestor of all living things, and this protein experienced gene duplication, differentiation, and eventually developed independently into members of Cter-Sad1-UNC proteins and Mid-Sad1-UNC proteins in prokaryotes. These two types of SUNs acquired a coiled-coil domain and a TMH domain at the N-terminus (Cter-SUNs) or C-terminus (Mid-SUNs) of the SUN domain during the emergence of eukaryotes. Most Cter-SUNs were well conserved in eukaryotes and developed SUN1 except for a few yeasts in which this became MPS3. Prior to the emergence of angiosperms, Mid-SUNs were further duplicated and gradually evolved into SUN3 and SUN5. While we have a general idea of when these events occurred, more detailed biological functions of SUN5 and SUN3 still need to be determined. Further experiments need to be performed to reveal functional divergence of Mid-SUN in higher plants, and this will improve our understanding of the diversification and functional evolution of the whole SUN family.

## Data Availability Statement

The original contributions presented in the study are included in the article/Supplementary Material, further inquiries can be directed to the corresponding author/s.

## Author Contributions

LY, WC, and YZ conceived the research and designed the experiments. LY performed the experiments. JP, SZ, QL, XW, BL, SF, and CL participated in the experiments. LY, WC, and YZ analyzed the data and experiment results. LY and YZ wrote the manuscript. All authors read and approved the final manuscript.

## Conflict of Interest

The authors declare that the research was conducted in the absence of any commercial or financial relationships that could be construed as a potential conflict of interest.

## Footnotes

1 https://sep2019-protists.ensembl.org/index.html

2 http://fungi.ensembl.org/

3 https://phytozome.jgi.doe.gov/

4 http://www.cottonfgd.org/

5 https://www.arabidopsis.org/

6 http://bacteria.ensembl.org/

7 http://meme-suite.org/tools/meme

8 http://gsds.cbi.pku.edu.cn/

9 http://www.cbs.dtu.dk/services/TMHMM/

10 https://embnet.vital-it.ch/software/TMPRED_form.html

11 https://embnet.vital-it.ch/software/COILS_form.html

12 http://www.timetree.org/

13 https://cottonfgd.org/analyze/

14 http://cotton.zju.edu.cn/

15 http://www.gene-clime.org/
