# Supplementary material for: Evolution and Functional Divergence of SUN Genes in Plants
Source: Front Plant Sci. 2021 Mar 8;12:646622. doi: 10.3389/fpls.2021.646622 (PMC7982736; doi:10.3389/fpls.2021.646622)

Additional file 1. CLIME (CLustering by Inferred Models of Evolution) Analysis of 4 Arabidopsis *AtSUN* genes.

Overview of Evolutionarily Conserved Modules (ECMs)

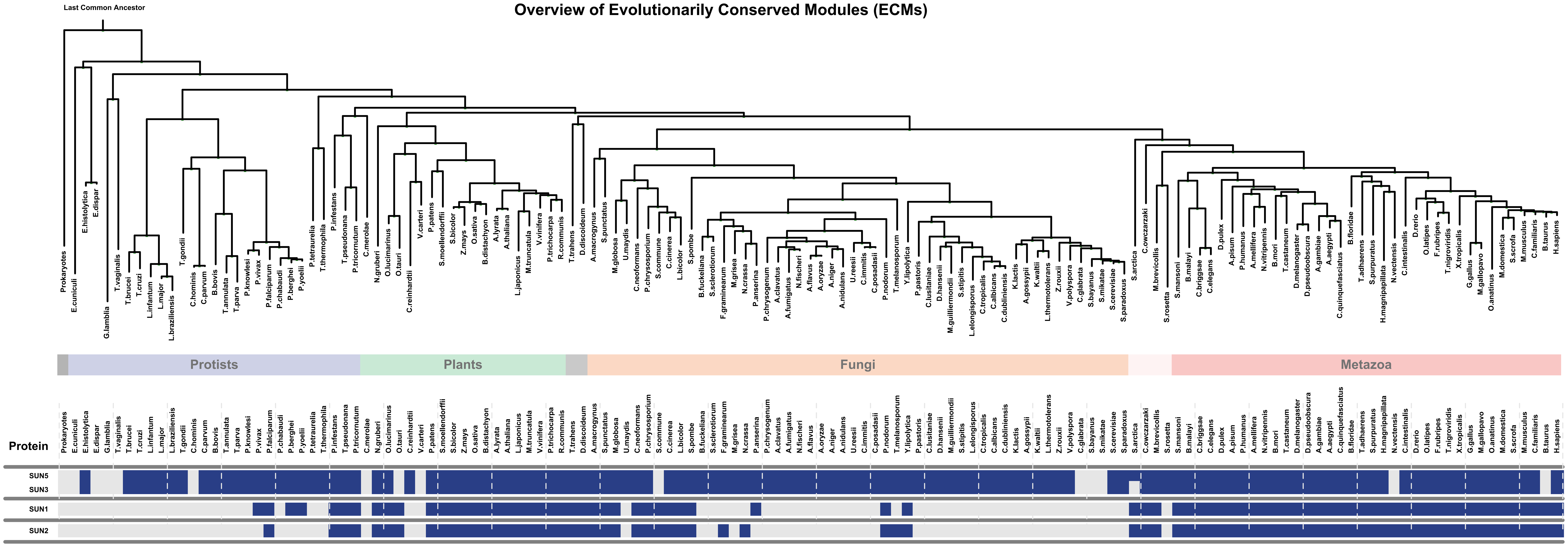

Supplement: Supplementary file 1 [file Presentation_1.pdf]
