# Supplementary figures and images for: Evolution and Functional Divergence of SUN Genes in Plants
Source: Front Plant Sci. 2021 Mar 8;12:646622. doi: 10.3389/fpls.2021.646622 (PMC7982736; doi:10.3389/fpls.2021.646622)

## Overview of Evolutionarily Conserved Modules (ECMs)

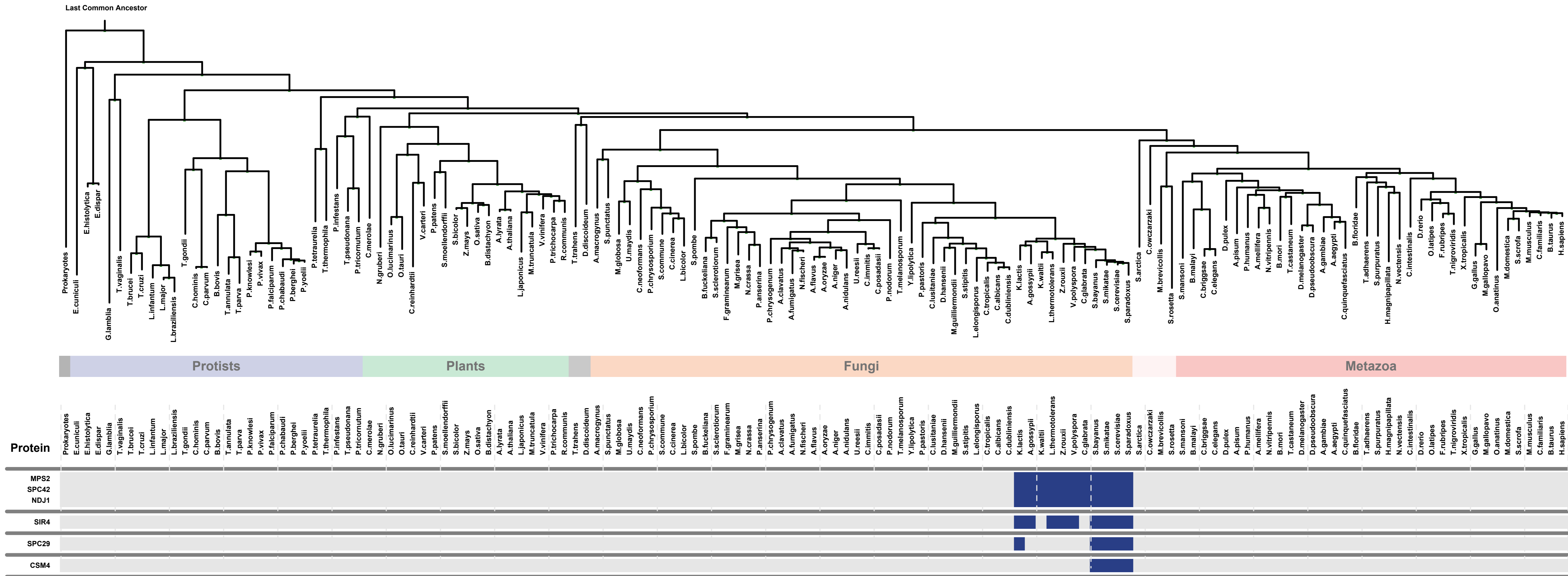

Supplement: Supplementary file 3 [file Presentation_3.pdf]
