# Supplementary material for: Evolution and Functional Divergence of SUN Genes in Plants
Source: Front Plant Sci. 2021 Mar 8;12:646622. doi: 10.3389/fpls.2021.646622 (PMC7982736; doi:10.3389/fpls.2021.646622)
Supplement: Supplementary file 5 [file Data_Sheet_2.pdf]

Supplementary Material

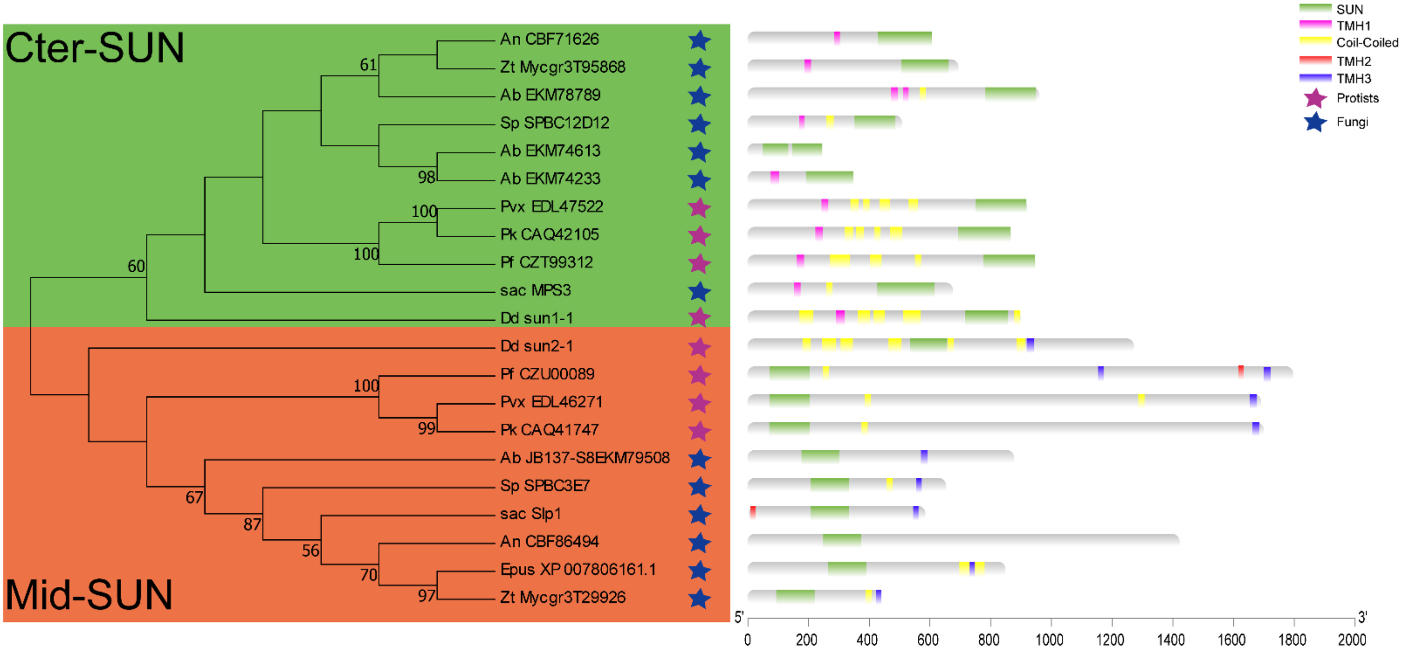

Supplementary Figure S1. Conserved domain organizations and Phylogenies analysis of *SUN* genes in representative protists and fungi.

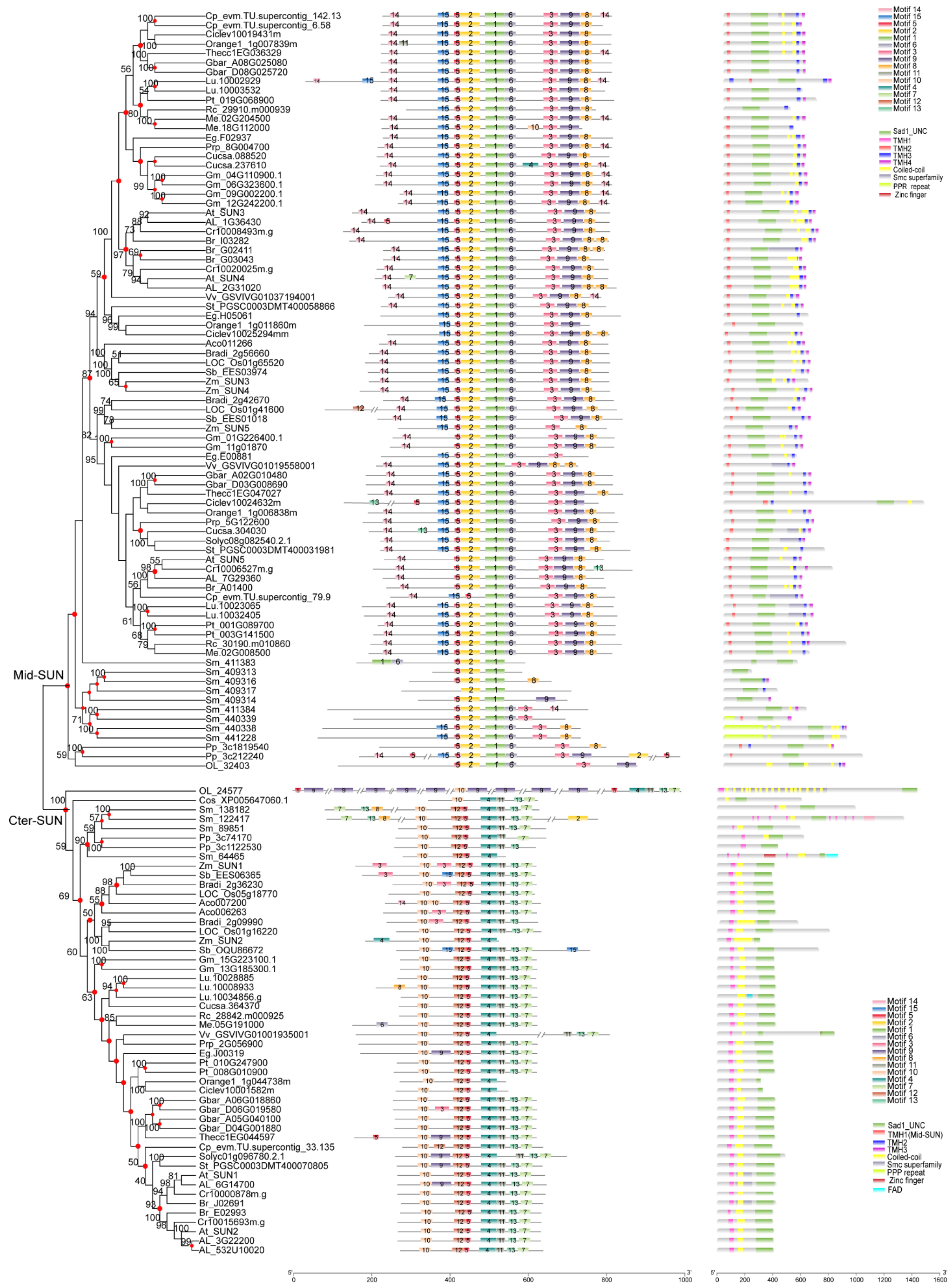

Supplementary Figure S2. Phylogeny and sequence analysis of plant SUN genes.

Tree topology generated by MEGA-X was demonstrated here. ML bootstrap values above 50% are shown. The red dots at internal nodes denote where gene duplication events have occurred. SUN genes are separated into Cter-SUN and Mid-SUN. Conserved motifs are displayed in the different colored boxes with various numbers on the middle. Protein domain organizations are shown as colored boxes on the right.

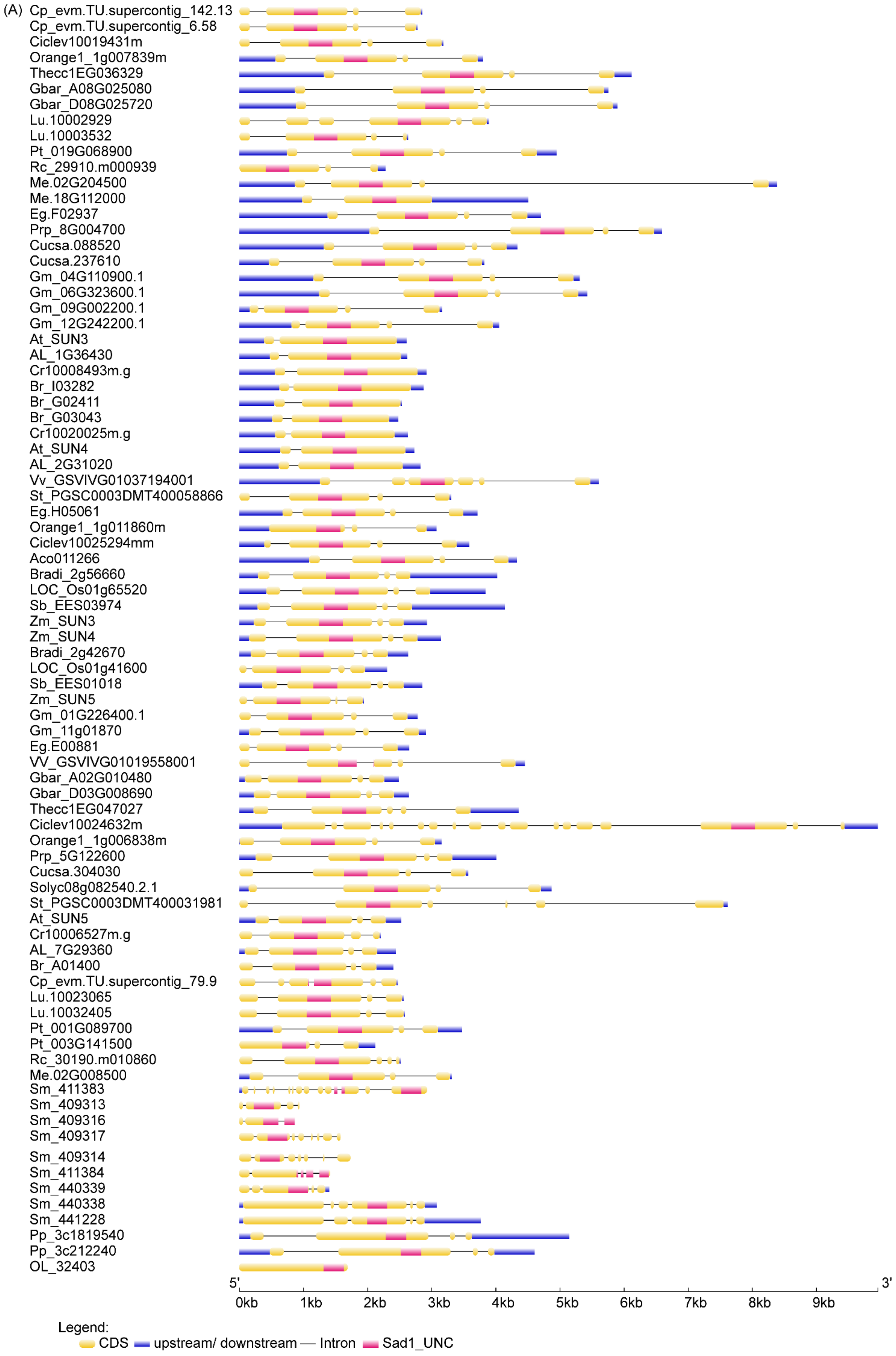
(A)
Cp_evm.TU.supercontig_142.13
Cp_evm.TU.supercontig_6.58
Ciclev10019431m
Orange1_1g007839m
Thecc1EG036329
Gbar_A08G025080
Gbar_D08G025720
Lu.10002929
Lu.10003532
Pt_019G068900
Rc_29910.m000939
Me.02G204500
Me.18G112000
Eg.F02937
Prp_8G004700
Cucsa.088520
Cucsa.237610
Gm_04G110900.1
Gm_06G323600.1
Gm_09G002200.1
Gm_12G242200.1
At_SUN3
AL_1G36430
Cr10008493m.g
Br_I03282
Br_G02411
Br_G03043
Cr10020025m.g
At_SUN4
AL_2G31020
Vv_GSVIVG01037194001
St_PGSC0003DMT400058866
Eg.H05061
Orange1_1g011860m
Ciclev10025294mm
Aco011266
Bradi_2g56660
LOC_Os01g65520
Sb_EES03974
Zm_SUN3
Zm_SUN4
Bradi_2g42670
LOC_Os01g41600
Sb_EES01018
Zm_SUN5
Gm_01G226400.1
Gm_11g01870
Eg.E00881
VV_GSVIVG01019558001
Gbar_A02G010480
Gbar_D03G008690
Thecc1EG047027
Ciclev10024632m
Orange1_1g006838m
Prp_5G122600
Cucsa.304030
Solyc08g082540.2.1
St_PGSC0003DMT400031981
At_SUN5
Cr10006527m.g
AL_7G29360
Br_A01400
Cp_evm.TU.supercontig_79.9
Lu.10023065
Lu.10032405
Pt_001G089700
Pt_003G141500
Rc_30190.m010860
Me.02G008500
Sm_411383
Sm_409313
Sm_409316
Sm_409317
Sm_409314
Sm_411384
Sm_440339
Sm_440338
Sm_441228
Pp_3c1819540
Pp_3c212240
OL_32403
5'
3'
0kb
1kb
2kb
3kb
4kb
5kb
6kb
7kb
8kb
9kb
Legend:
CDS
upstream/ downstream
Intron
Sad1_UNC

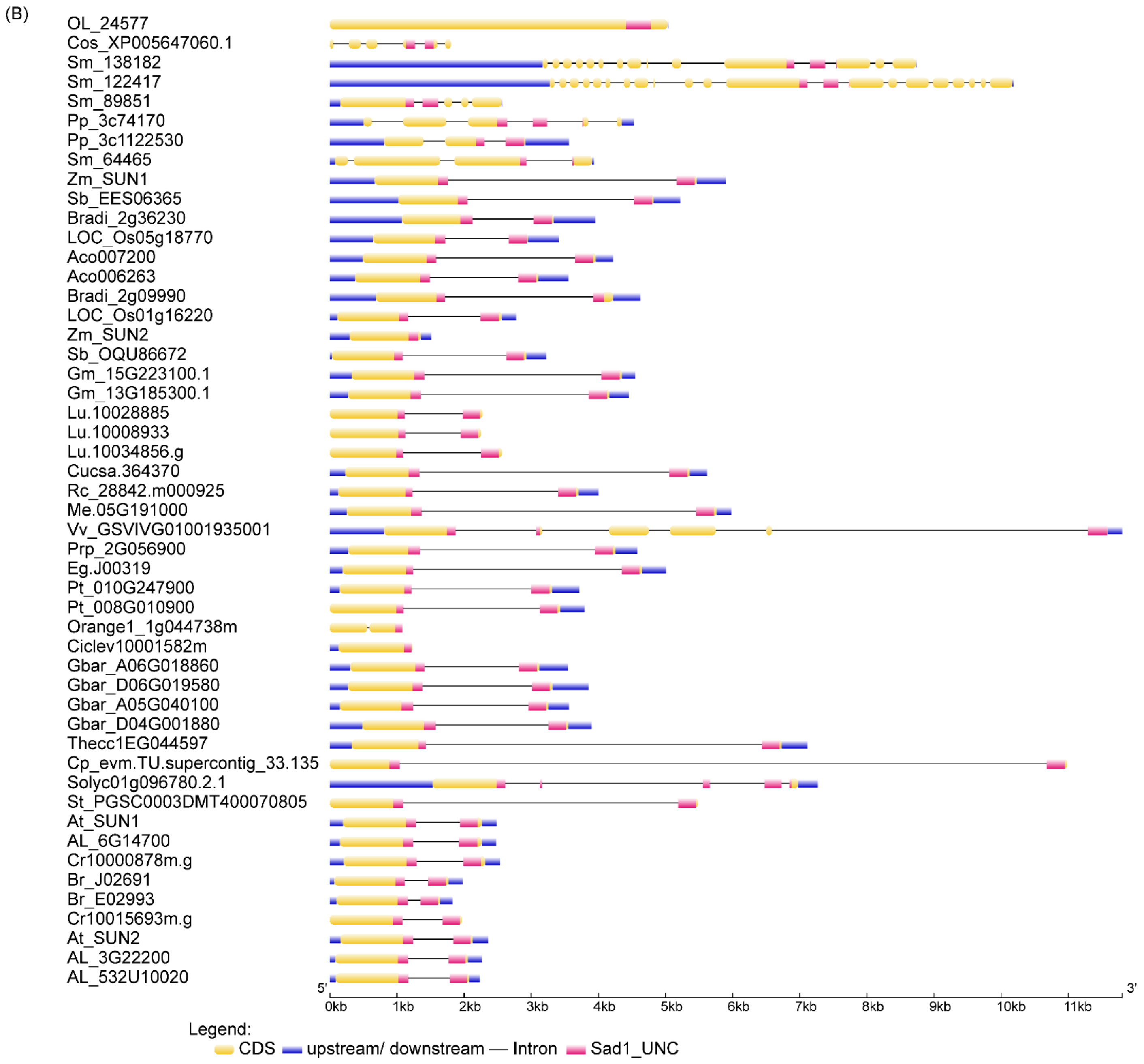

Supplementary Figure S3. Gene structure analysis of Mid-SUN genes (A) and Cter-SUN genes (B) in plant with the same genes as Figure S2.

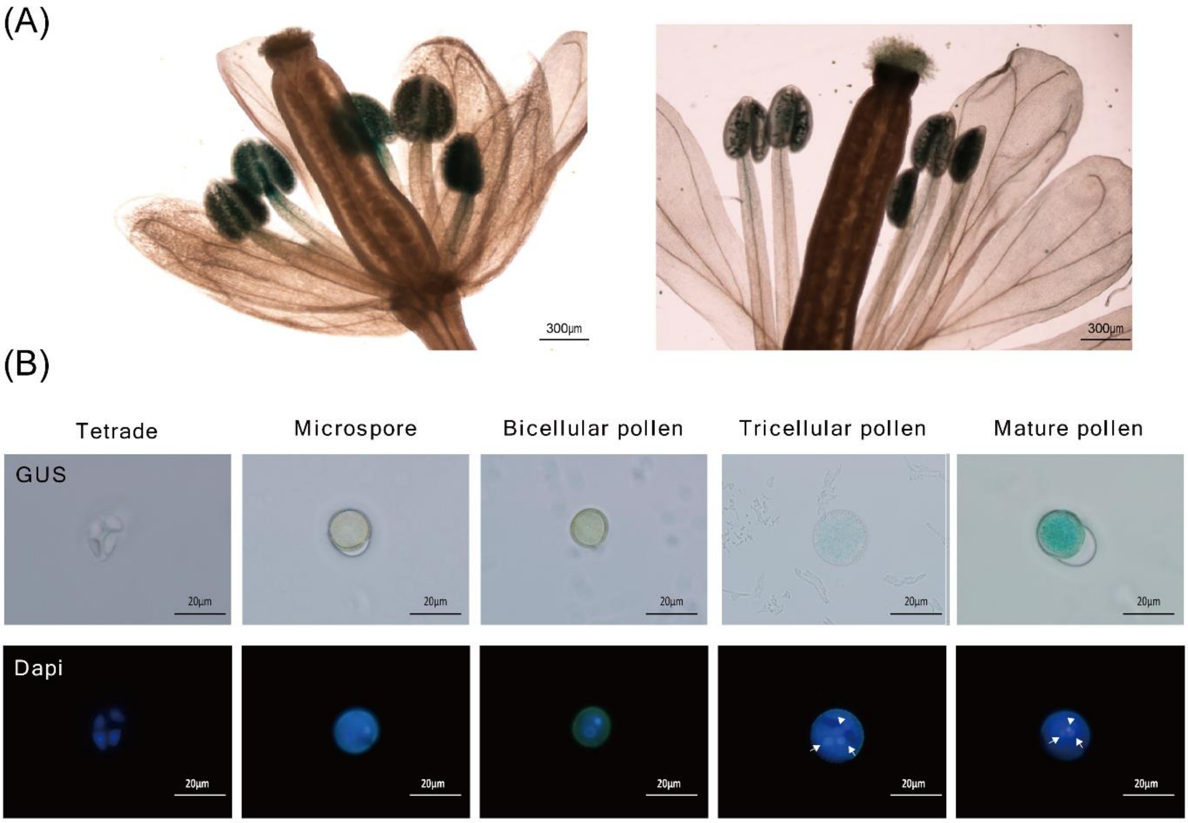

Supplementary Figure S4. Analysis of *AtSUN5* promote activity at different tissues.

(A) In mature anthers of plant, strong GUS staning was observed in mature pollen grains without the powder disperses (indicated on the left). GUS staining was also detected in mature pollen and stigma, when the male flower blossoms the powder disperses (indicated on the right).

(B) Blue GUS staining at different stages of pollen development.

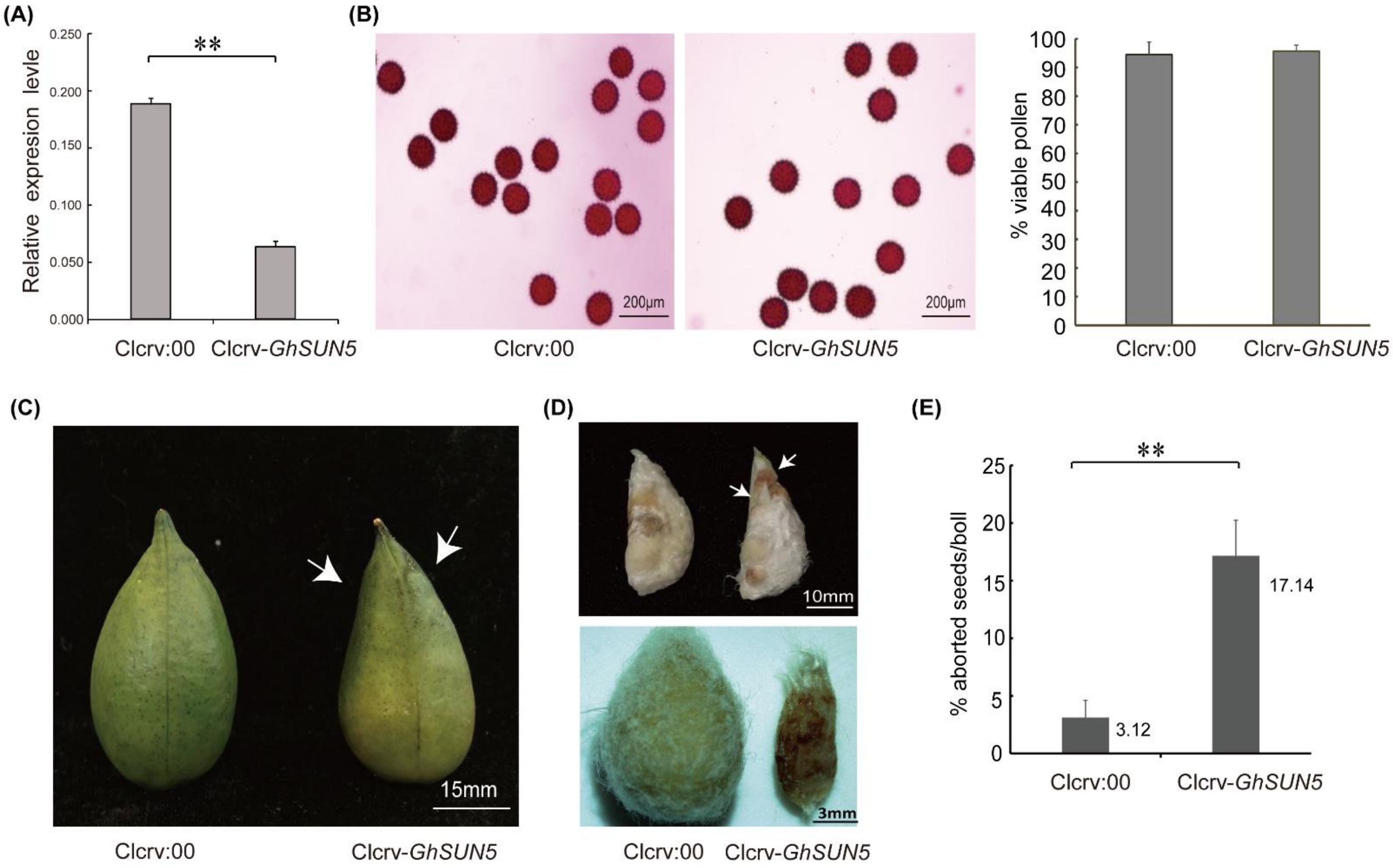

Supplementary Figure S5. Phenotyped of virus-induced gene silencing (VIGS) plants and suppression of endogenous transcripts in upland cotton.

(A) qRT-PCR analysis was used to examine the transcript levels of *GhSUN5* in mature pollen from plants infected with Clcrv-00 or Clcrv-*GhSUN5*. Data are means of three replicates. Significant differences between means were determined using Student's t-test: **P<0.01. (B) Pollen activity observation (left) and measurement (right) in Clc-*GhSUN5* plants and Clcrv-00 plants. (C)- (D) Phenotypes of Clcrv-00 and Clcrv-*GhSUN5* plants. Abnormal cotton bolls and aborted seeds were observed in Clcrv-*GhSUN5* compared with Clcrv-00. White arrow indicated cotton boll or seeds were aborted. (E) Percentage of aborted seeds per boll in Clcrv: 00 and Clcrv-*GhSUN5* plants. Data are shown as means in pollen activity testing and seed abortion determination (numbers of flower or boll >30) and statistically analyzed by using Student's t-test: **P<0.01.

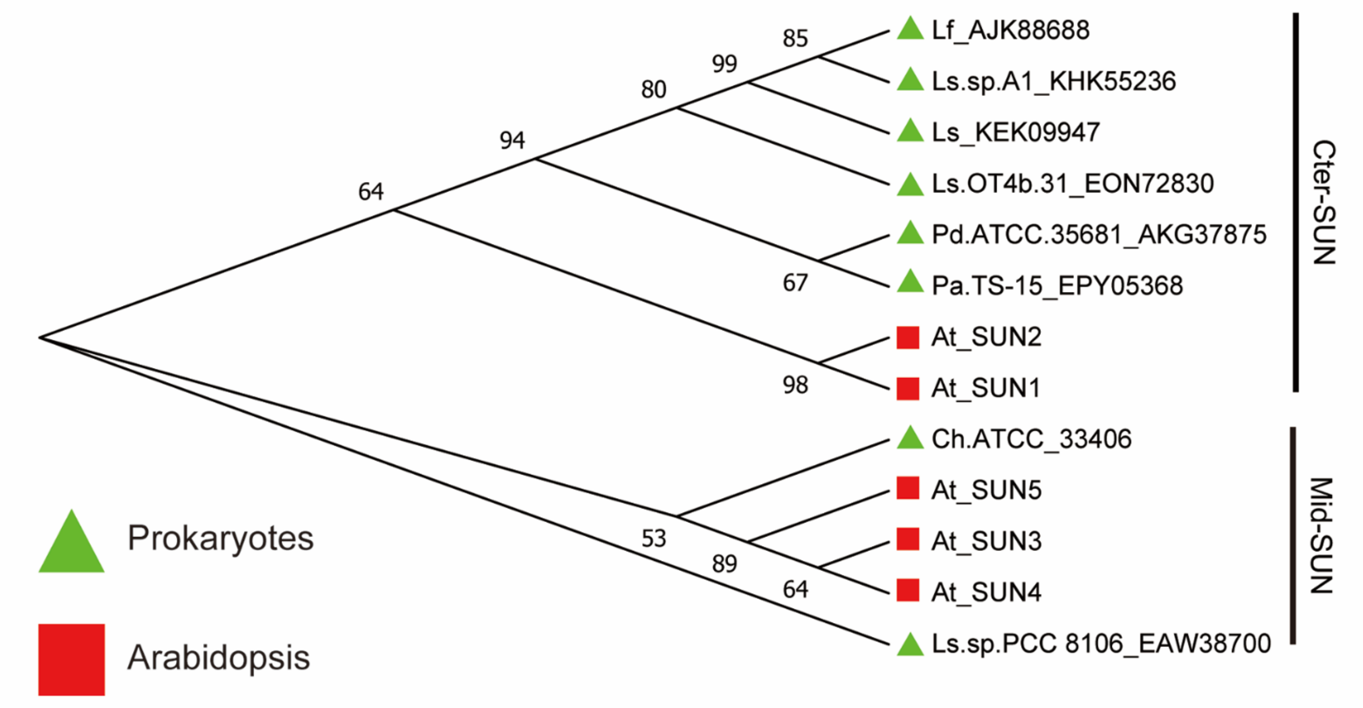

Supplementary Figure S6 A maximum likelihood (ML) tree showing the evolution of *SUN* genes in prokaryotes and plant. Squence from several prokaryotes and *Arabidopsis* are used.

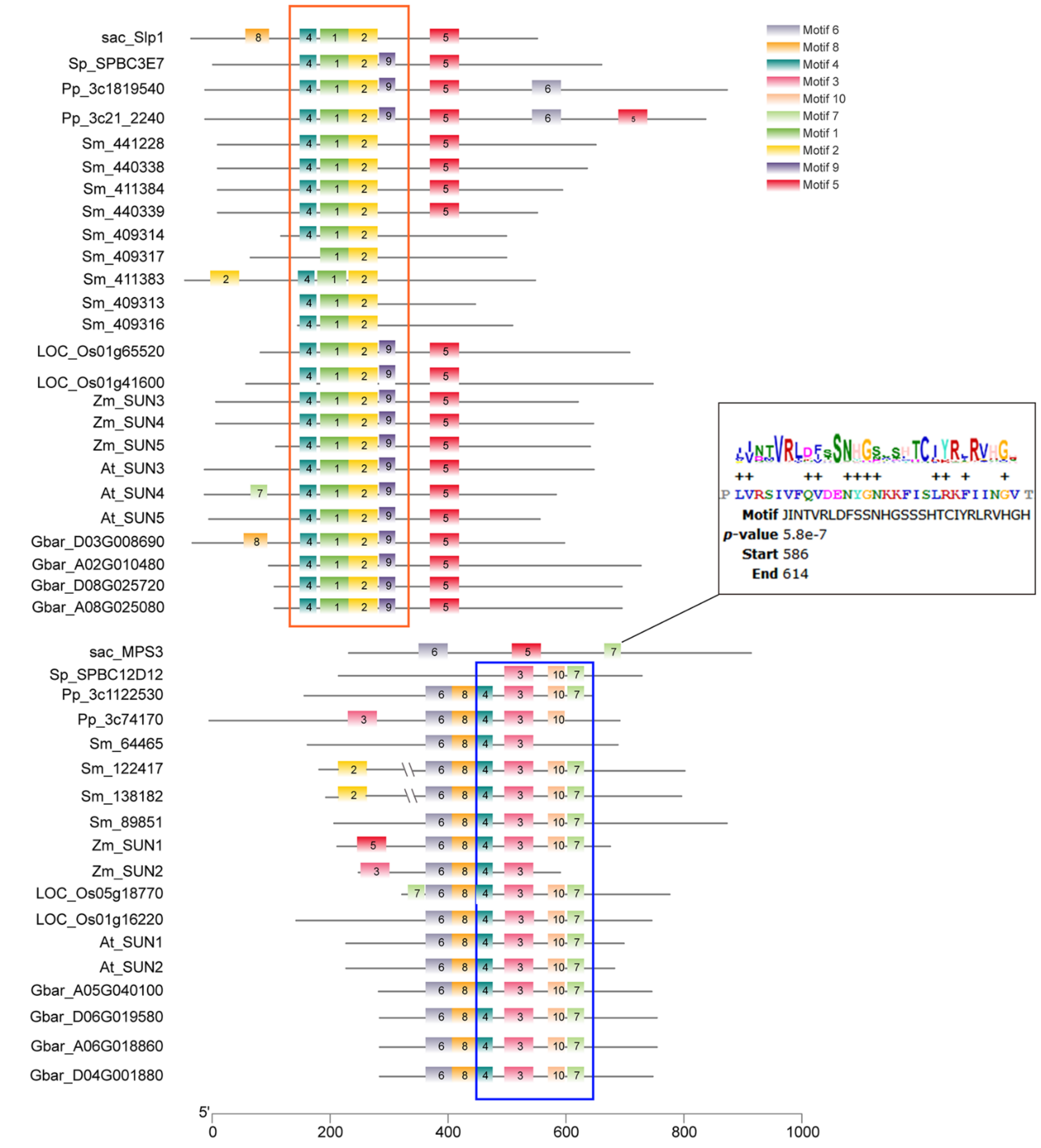

Supplementary Figure S7 Converted motif analyses of Cter-SUN and Mid-SUN in representative species. Orange rectangular box denote M-SUN domain. Blue rectangular box denote C-SUN domian.

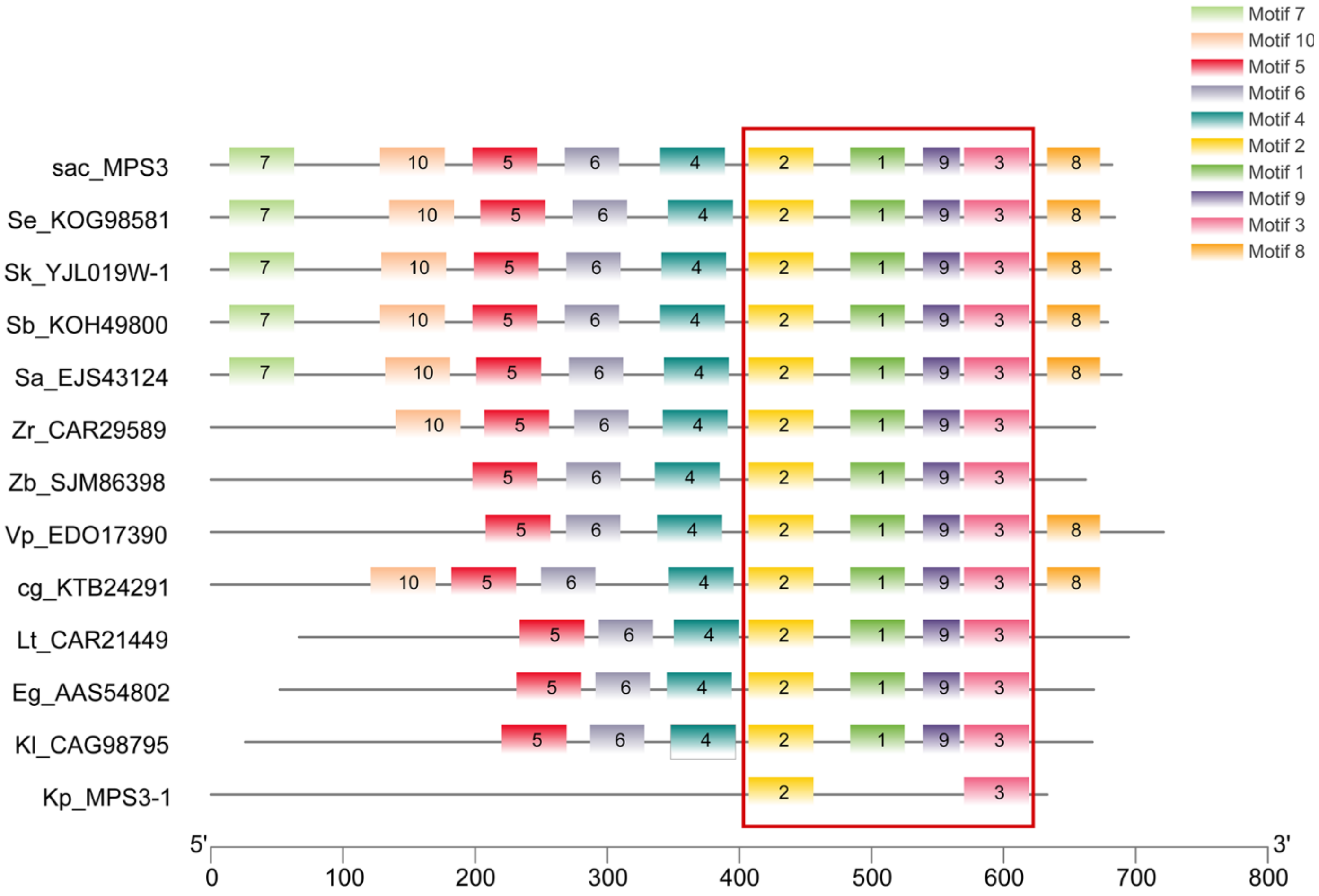

Supplementary Figure S8 Converted motif analyses of MPS3 in several yeast. Red rectangular box denote C-SUN domain which is different from other eukaryotic's
